# Altered type I collagen networking in osteoporotic human femoral head revealed by histomorphometric and Fourier transform infrared imaging correlated analyses

**DOI:** 10.1002/biof.1870

**Published:** 2022-06-06

**Authors:** Caterina Licini, Valentina Notarstefano, Saverio Marchi, Giorgia Cerqueni, Gabriela Ciapetti, Chiara Vitale‐Brovarone, Elisabetta Giorgini, Monica Mattioli‐Belmonte

**Affiliations:** ^1^ Department of Clinical and Molecular Sciences (DISCLIMO) Università Politecnica delle Marche Ancona Italy; ^2^ Department of Applied Science and Technology Politecnico di Torino Torino Italy; ^3^ Department of Life and Environmental Sciences Università Politecnica delle Marche Ancona Italy; ^4^ Laboratory of Nanobiotechnology (NaBi) IRCCS Istituto Ortopedico Rizzoli Bologna Italy

**Keywords:** bone ECM, FTIRI, immunohistochemistry, non‐collagenous proteins, osteoporosis, type I collagen

## Abstract

Bone homeostasis is the equilibrium between organic and inorganic components of the extracellular matrix (ECM) and cells. Alteration of this balance has consequences on bone mass and architecture, resulting in conditions such as osteoporosis (OP). Given ECM protein mutual regulation and their effects on bone structure and mineralization, further insight into their expression is crucial to understanding bone biology under normal and pathological conditions. This study focused on Type I Collagen, which is mainly responsible for structural properties and mineralization of bone, and selected proteins implicated in matrix composition, mineral deposition, and cell‐matrix interaction such as Decorin, Osteocalcin, Osteopontin, Bone Sialoprotein 2, Osteonectin and Transforming Growth Factor beta. We developed a novel multidisciplinary approach in order to assess bone matrix in healthy and OP conditions more comprehensively by exploiting the Fourier Transform Infrared Imaging (FTIRI) technique combined with histomorphometry, Sirius Red staining, immunohistochemistry, and Western Blotting. This innovatory procedure allowed for the analysis of superimposed tissue sections and revealed that the alterations in OP bone tissue architecture were associated with warped Type I Collagen structure and deposition but not with changes in the total protein amount. The detected changes in the expression and/or cooperative or antagonist role of Decorin, Osteocalcin, Osteopontin, and Bone Sialoprotein‐2 indicate the deep impact of these NCPs on collagen features of OP bone. Overall, our strategy may represent a starting point for designing targeted clinical strategies aimed at bone mass preservation and sustain the FTIRI translational capability as upcoming support for traditional diagnostic methods.

**Abbreviations**
μCT: micro‐computed tomography
AGEs: advanced glycation ends
B.Ar./T.Ar.: bone area
BSP‐2: bone sialoprotein 2
Co.Th.: cortical thickness
COL1A1: type I collagen alpha 1 chain
COL1A2: type I collagen alpha 2 chain
DAB: 3,3′‐diaminobenzidine
DCN: decorin
ECM: extracellular matrix
FPA: focal plane array
FTIRI: Fourier transform infrared imaging
H: Healthy
HA: hydroxyapatite
HCA: hierarchical cluster analysis
LUT: look‐up tables
NCPs: non‐collagenous proteins
OBs: osteoblasts
OCN: osteocalcin
OCs: osteoclasts
ON: osteonectin
OP–: collagen‐poor areas
OP: osteoporosis
OP+: collagen‐rich areas
OPN: osteopontin
ROI: region of interest
TGF‐β: transforming growth factor beta
Tr.Th.: trabecular thickness
WB: Western blotting

## 1 INTRODUCTION

Throughout life, bone is subjected to a remodeling process that determines the quality and quantity of inorganic and organic components and ensures the maintenance of bone mass and architecture.

Osteoblasts (OBs) lay bone matrix promoting bone formation, while osteoclasts (OCs) regulate bone resorption through an intricate network of autocrine, paracrine, and endocrine factors, including the mutual signaling between OBs and OCs.^1^, ^2^, ^3^

Bone remodeling occurs at the endosteal surface in trabecular bone, and Haversian surface remodeling is the main mechanism at the cortical portion.^2^ An increase in the rate of bone remodeling and negative balance in OBs‐OCs coupling result in osteoporosis (OP), characterized by a decrease in bone mass, disrupted bone tissue microarchitecture, enhanced bone fragility, and increased fracture risk.^4^, ^5^

OP affects many bone extracellular matrix (ECM) proteins regulating bone physiology.^6^, ^7^ Type I Collagen is the most abundant protein in bone, constituting 90% of the matrix. The mature molecule comprises two 1a1 chains (COL1A1) and one 1a2 chain (COL1A2), held together by covalent bonds that ensure the triple helix conformation. Collagen triple helices interact with each other forming enzymatic cross‐links which guarantee their correct quaternary configuration as fibrils. In bone, Type I Collagen primarily provides structural and mechanical support, acting as a “scaffold” for the other ECM proteins, initial mineral deposition, and organization of crystal growth.^7^, ^8^ In OP subjects, Type I Collagen undergoes reduction of enzymatic cross‐links and a concurrent increase of Advanced Glycation End‐products (AGEs) generation which has structural and functional consequences.^5^, ^6^, ^9^

The Non‐Collagenous Proteins (NCPs) comprise about 180–200 different molecules that exert several roles in bone biology.^5^, ^10^ Type I Collagen and various NCPs interact with each other and are mutually regulated. For instance, Decorin (DCN), Osteonectin (ON), and Transforming Growth Factor‐ beta (TGF‐β) regulate Type I Collagen synthesis, assembly, and maturation, preventing its degradation and preserving its structure.^5^, ^11^, ^12^, ^13^ On the other hand, Type I Collagen is necessary to control the mineralization mediated by Osteocalcin (OCN), Bone Sialoprotein (BSP‐2), ON and Osteopontin (OPN). In particular, the association between OCN, OPN, and Type I Collagen is fundamental in bone mineralization.^5^, ^12^, ^14^, ^15^, ^16^

The ECM proteins' involvement in OP onset and maintenance has been investigated by mainly focusing on the identification of their changes as markers of disease in ovariectomized animal models or human biological fluids.^1^, ^5^, ^17^ However, an accurate assessment of ECM proteins distribution and expression in human OP bone and their potential action in the disease pathophysiology is far from being elucidated. In this work, we used an innovative and multidisciplinary approach, combining hyperspectral imaging analysis with morphological and molecular data to elucidate possible bone ECM alterations related to OP onset and maintenance. An appropriate protocol was developed to apply histomorphometry to 2D decalcified histological sections, which enabled the correlation of histomorphometric and immunohistochemical results obtained from the same samples. For studying the expression and the structure of Type I Collagen, crucial for good matrix deposition and mineralization, Sirius Red staining, immunohistochemistry, Western Blotting (WB), and Fourier Transform Infrared Imaging (FTIRI) spectroscopy were used.

FTIRI analysis of tissue samples empowered a morpho‐chemical correlation between the histological and spectroscopic data by the topographic detection of possible changes in the biochemical composition and/or conformation of the biomolecules of interest on the same tissue section.^18^, ^19^, ^20^, ^21^ As other techniques conventionally devoted to basic research, FTIRI represents an attractive molecular diagnostic modality for a future clinical translation, in addition to the traditional histopathological diagnosis.^22^, ^23^

Finally, we correlated bone architecture and Type I Collagen structural changes with the expression of NCPs like TGF‐β, DCN, OPN, BSP‐2, ON, OCN involved in bone structuring.

## 2 EXPERIMENTAL PROCEDURES

### 2.1 Ethics statement

Femoral head samples were collected after total hip arthroplasty at the Clinic of Orthopedics, Università Politecnica delle Marche, Ancona, Italy. All procedures followed were in accordance with the ethical standards of the responsible committee on human experimentation (institutional and national) and with the Helsinki Declaration of 1975, as revised in 2008. Informed consent was obtained from all patients for being included in the study. To all subjects was highlighted that the tissue used for the study represents the typical discard during the surgical procedures. Ethical compliance was obtained according to the Italian legislative decree May 14, 2019, n. 52, containing amendments to the legislative decree November 6, 2007, n. 200, implementing Directive 2005/28/EC, adopted in implementation of the delegation for the reorganization and reform of the legislation on clinical trials of medicinal products for human use. Patients had sufficient opportunity to ask questions and consider their choice.

### 2.2 Sample collection and processing

Seven femoral heads, four from osteoporotic (OP) patients and three from healthy (H) subjects (enrolled subjects were all female, OP 80.5 ± 2.52 years vs. H 70.75 ± 22.2 years, *p* = 0.999), immediately washed by ice‐cold PBS 1X and then stored at −80 °C until use. Blood samples for serum osteocalcin detection were also collected.

For histological and immunohistochemical investigations, tissue specimens were obtained from the femoral heads by using chisel and hammer, fixed in 4% paraformaldehyde for 48 h at 4 °C, washed in phosphate buffer pH 7.4 for 48 h, and decalcified by Biodec R for 6 h. After decalcification, samples were washed in PBS 1X and dehydrated by increasing alcohol grade and xylene before paraffin embedding.

For WB analysis, bone tissues were collected using a chisel and hammer and processed as previously described.^24^ Briefly, bone specimens were immersed in a saline solution at pH 7.2 (0.05 M NaCl, 0.02 M NaH_2_PO_4_, 0.03 M Na_2_HPO_4_) with protease inhibitors, sonicated in an ultrasonic bath for 1 min and interposed with 1 min in ice, for five cycles overall, to degrease and remove the remaining soft tissues and cells. Samples were then powdered by mortar and pestle in liquid nitrogen and 100 mg of bone powder resuspended for Type I Collagen, TGF‐β, DCN, OPN, and BSP‐2 were dissolved in 1 ml of TRIzol reagent and processed as previously described.^24^ For Osteocalcin, 1 g of bone powder was resuspended in G solution (4 M Guanidine Hydrochloride in 0.05 M Tris–HCl pH 7.4 and protease inhibitors) and treated as previously reported.^24^

### 2.3 Histological staining and analyses

#### 2.3.1 Hematoxylin and eosin staining and histomorphometry

Three series of five 8‐μm‐thick sections were cut every 100 mm from each sample, ensuring replicate sections with different cortical bone portions and trabeculae along 400 mm depth before staining with Hematoxylin & Eosin.^25^ For the morphological investigation, images were taken at different magnifications, while, for histomorphometry, images were captured at 10X magnification from the whole section and stitched by MosaicJ plugin for Fiji software. Due to the difficulty in determining if the distances and number of the trabeculae could be a cutting artifact,^26^ reconstructed entire tissue sections were analyzed to calculate bone area fraction (B.Ar./T.Ar.), cortical (Co.Th.) and trabecular (Tr.Th.) thickness. Fiji software and BoneJ plugin were used for the image processing.

#### 2.3.2 Sirius red staining and semi‐quantitative analysis

Six‐μm‐thick sections from each sample were stained by Sirius Red and analyzed by brightfield and fluorescent microscopy to evaluate collagen distribution.^27^, ^28^ For the semi‐quantitative analysis, eight images (four from cortical bone and four from trabecular bone) at 40X magnification from each sample were obtained by fluorescent microscopy, focusing on the red channel (Collagen), and analyzed by Fiji software. Briefly, each image was converted to 8‐bit and then to a 16‐colors Lookup Table (LUT). After assigning thresholds, bone tissue was selected as Regions of Interest (ROI) and the area percentage of Collagen was calculated.

### 2.4 FTIR imaging measurements and data analysis

FTIR Imaging measurements were performed by a Bruker INVENIO interferometer coupled with a Hyperion 3000 Vis‐IR microscope. A liquid nitrogen cooled Focal Plane Array (FPA) detector which allows acquiring IR maps, was used.

From each paraffin‐embedded sample, three thin sections (5 μm thick) were cut, immediately deposited onto CaF_2_ optical windows (13 mm diameter, 1 mm thick), and let air dry for 30 min. The microphotograph of each section was acquired by using a 15× condenser objective. Specific areas containing portions of cortical and trabecular bones were selected for the acquisition of IR maps in transmission mode in the 4000–800 cm^−1^ spectral range; each map was 164 × 164 mm in size and contained 4096 pixel/spectra with a spatial resolution of 2.56 × 2.56 mm. IR maps were then preprocessed as follows: atmospheric water vapor and carbon dioxide correction (Atmospheric Compensation routine, OPUS 7.5, Bruker Optics, Ettlingen, Germany); interpolation in the 1800–900 cm^−1^ spectral range, to avoid paraffin contribution, and vector normalization in the same interval (Vector Normalization routine, OPUS 7.5, Bruker Optics, Ettlingen, Germany). False color images representing the topographical distribution of collagen within the mapped area were created by integrating the preprocessed IR maps under the 1300–1183 cm^−1^ spectral range, including Amide III band. Preprocessed IR maps were also submitted to Hierarchical Cluster Analysis (HCA) by Euclidean distances and Ward's linkage method (software CytoSpec v. 2.00.01). Based on HCA maps, the clusters representative of collagen were identified on all sections and their corresponding spectra extracted. As regards H samples, the average spectrum (centroid) was calculated, together with the average ± SD spectra (Averaging routine, OPUS 7.5, Bruker Optics, Ettlingen, Germany). The same procedure was applied on OP samples, but in this case, two sets of spectra were identified, representative of collagen‐rich areas (OP+) and collagen‐poor ones (OP–).

All average spectra and average ± SD spectra were then curve fitted in the 1365–1130 cm^−1^ region. The number and position of all the underlying bands were evaluated by Second Derivative minima analysis and fixed during the fitting procedure with Gaussian functions (GRAMS/AI 9.1, Galactic Industries, Inc., Salem, New Hampshire). In order to assess the relative amount and composition of collagen among groups, the integrated area of specific underlying bands were used to calculate the following band area ratios: Coll/Tot (ratio between the sum of the peaks centered at 1284, 1240, and 1204 cm^−1^, and the sum of all the peaks identified in the 1365–1130 cm^−1^ spectral region); 1340/1160; 1320/1160; 1284/1160; 1264/1160; 1240/1160; 1204/1160, and Random/Folded (ratio between the integrated area of the band centered at 1264 cm^−1^ and the sum of the integrated areas of the peaks centered at 1320, 1284, and 1240 cm^−1^). The choice of ratioing integrated areas against the area of the peak centered at 1160 cm^−1^ (representative of C‐OH moiety of carbohydrates), which did not vary among groups, was due to avoid changes in height and areas of peaks, ascribable to local thickness variations.

### 2.5 Immunohistochemistry and immunostaining evaluation

For immunohistochemical analysis, 6‐μm‐thick tissue sections were deparaffinized and rehydrated by xylene and a graded series of ethyl alcohols (from 100% to 50%), before incubation with 3% hydrogen peroxide for 30 min to block endogenous peroxidase activity. Antigen retrieval was performed in 0.05% Pepsin in HCl (for COL1A1), Citrate buffer pH 6 at 70°C for 10 min (for COL1A2, DCN, OPN, and OCN staining) and in 0.3% Tween 20 in PBS 1X at RT for 20 min (for TGF‐β, ON, and BSP‐2 staining). Sections were then incubated overnight at 4°C with primary antibodies (Table 2S). Antigens were visualized by Envision Dako REAL™ EnVision™ Detection System. Sections were counterstained with Mayer's Hematoxylin, dehydrated, and mounted in Biomount HM.

Immunostaining was semi‐quantitatively evaluated by Fiji software, obtaining a percentage of the stained area. Concisely, six different images (three from cortical bone and three from trabecular bone) at ×20 magnification from each tissue sample were subjected to color deconvolution––to subtract background and analyze only the 3,3′‐Diaminobenzidine (DAB) staining. The stained area percentage was obtained as described above. Different minimum and maximum thresholds were assigned for each investigated antibody, to standardize results and valorize each staining.

To be sure to evaluate only ECM proteins, we excluded cells and osteocyte lacunae in the immunostaining assessment.

### 2.6 Western blotting

Total protein concentration was calculated by DC protein assay, and protein samples were prepared to load 20 μg of protein for each sample. To analyze TGF‐β, IGF‐1, DCN, OPN, BSP‐2, ON, and OCN expression, samples were prepared using NuPAGE™ LDS Sample Buffer and fractionated in NuPAGE™ 4–12% Bis‐Tris Protein Gels. To evaluate Type I Collagen expression, samples were prepared using Novex™ Tris‐Glycine SDS Sample Buffer and fractionated in Novex™ WedgeWell™ 8% Tris‐Glycine Gels. Proteins were electrophoretically transferred to 0.2 μm nitrocellulose membranes.

Membranes were incubated with 5% milk in Tris‐Buffered Saline with 0.1% Tween 20 (TBS‐T) to block non‐specific sites and then with primary antibodies in TBS‐T at 4 °C overnight (Table 2S).

After washes with TBS‐T, the membranes were incubated with secondary antibody anti‐mouse or anti‐rabbit conjugated with horseradish peroxidase. Detection of antibody binding was performed with Pierce ECL Western Blotting Substrate, and images were acquired with Alliance Mini HD9. Densitometric analysis was performed with Fiji software. Total protein normalization was calculated on Ponceau S stain, as no housekeeping proteins can be detected in decellularized bone tissue.^24^ Membrane was not stripped between antibody incubations to avoid protein loss.

### 2.7 Statistical analysis

Statistical analyses were performed using GraphPad Prism 7 software. Data were presented as mean ± SD. Significance was accepted when the P‐value was <0.05. Mann–Whitney test was used to compare serum OCN, histomorphometrical, histochemical and WB data between H and OP subiects. Pearson's correlation was applied to test the relationship between FTIRI parameters and Sirius Red staining, between the histochemical staining and the expression of each investigated protein and among the expression detected in WB for each investigated protein. Significant differences among experimental groups were evaluated by means of factorial analysis of variance (one‐way ANOVA), followed by Tukey's multiple comparisons test, for data collected from FTIRI and Sirius Red staining.

## 3 RESULTS

### 3.1 Histomorphometric analysis on 2D decalcified histological sections revealed altered bone architecture in OP tissue

**FIGURE 1:**
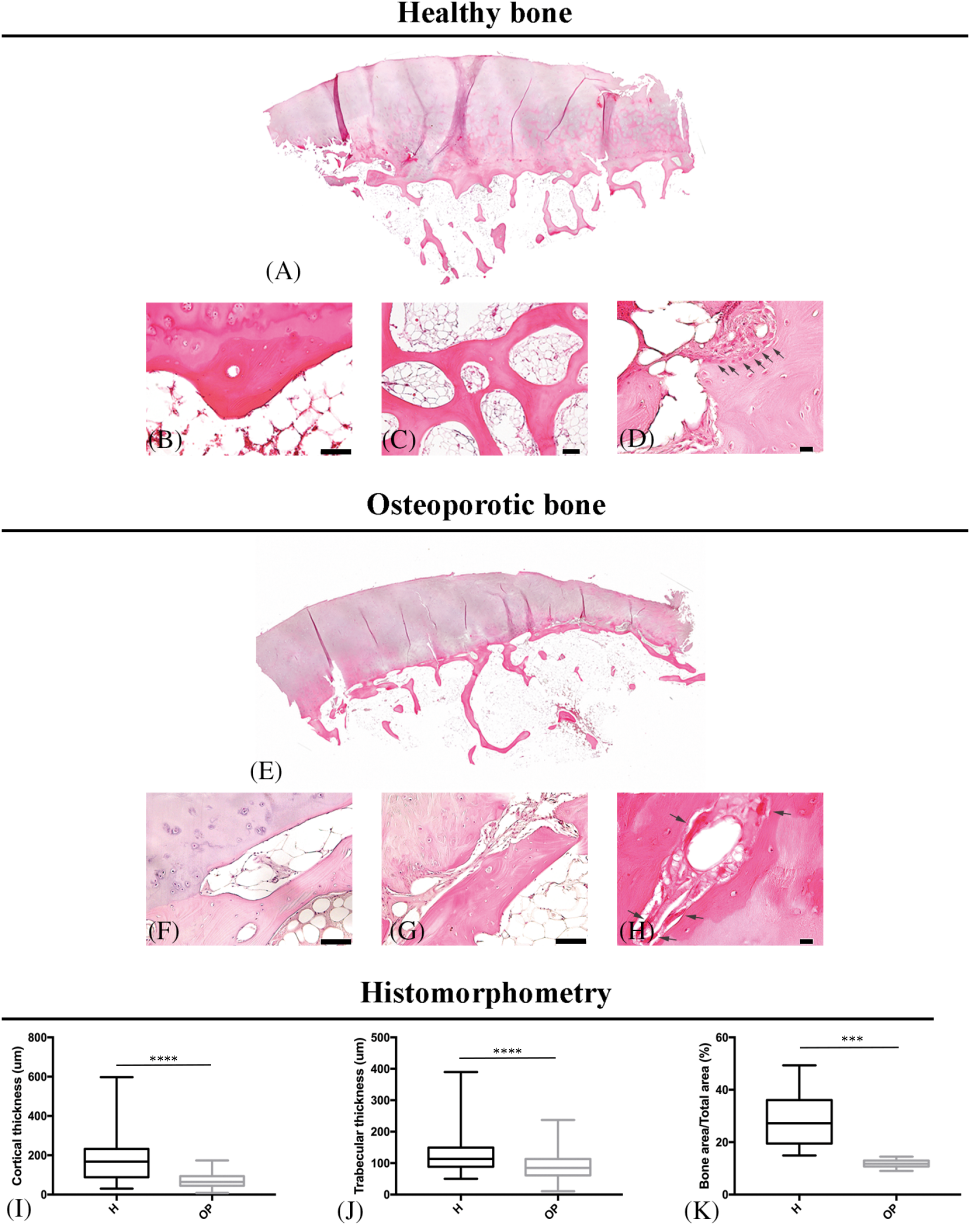
Morphological features of hematoxylin & eosin‐stained bone tissues from healthy (A–D) and osteoporotic (E–H) subjects and histomorphometrical evaluation (I–K). (A) Mosaic of bone tissue section images; (B) osteon in cortical bone (×20 magnification, 50 μm scale bar); (C) trabecular bone interconnections (×10 magnification, 100 μm scale bar); (D) bone formation zone in cortical bone (black arrows indicate OBs; ×40 magnification, 10 μm scale bar); (E) mosaic of bone tissue section images; (F–G) depletion and “trabecularization” of cortical bone (×20 magnification, 50 μm scale bar); (H) bone resorption zone in cortical bone (black arrows indicate OCs; ×40 magnification, 10 μm scale bar); graphical representation of (I) cortical thickness, (H) trabecular thickness and (K) bone area values in healthy (H) and osteoporotic (OP) bone

Morphological features of H and OP bone tissues are depicted in Figure 1.

Healthy sections showed a regular conformation of subchondral bone, with a uniform thickness of cortical bone, as well as orderly packaged trabeculae, with a well‐structured trabecular interconnection (Figure 1A–C). Several bone deposition zones were also found (Figure 1D).

On the contrary, the cortical bone was thin and discontinuously arranged under the articular cartilage in OP bone (Figure 1E). In particular, cortical “trabecularization” in numerous parts (Figure 1E–G), disordered trabeculae not uniformly distributed under the lines of force (Figure 1E), and several resorption zones in the cortical area were observed. (Figure 1H).

Histomorphometric measurements confirm that the cortical (Co.Th.) and trabecular thickness (Tr.Th.) were lower in OP than in H tissues (Figure 1I, J) and that the bone area fraction (B.Ar./T.Ar.) was significantly higher in H than in OP bones. (Figure 1K).

### 3.2 Type I collagen structure and localization were altered in defined regions of OP bone with no effects on the total protein amount

**FIGURE 2:**
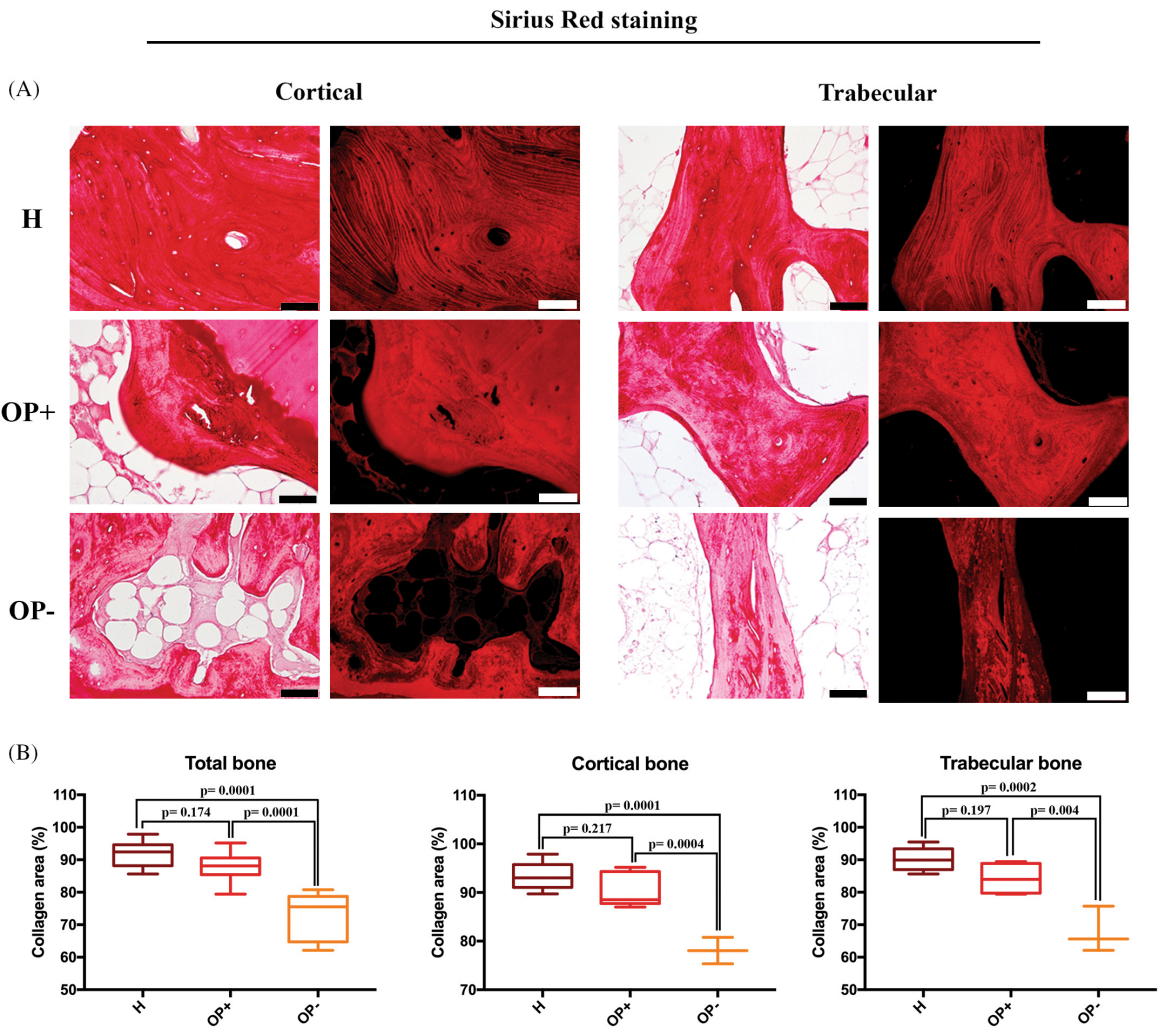
Type I collagen expression in healthy (H) and osteoporotic (OP) bone. (A) Brightfield and fluorescence images of Sirius red‐stained cortical and trabecular bone (×20 magnification, 50 μm scale bar) and (B) graphical representation of the semi‐quantification for healthy samples (H), collagen‐enriched zones (OP+) and collagen‐impoverished zones (OP–) in osteoporotic samples

In H bone, Sirius Red staining demonstrated well‐ordered collagen fibers. Both brightfield and fluorescence images underlined a marked stain along the lamellae in the cortical or trabecular bone. (Figure 2a) In OP sections, we observed zones with different collagen distribution, some comparable to the healthy samples, others with discontinuous staining. For this reason, we decided to analyze Sirius Red staining distinguishing in collagen‐rich zones, called OP+, and collagen‐poor zones called OP–. (Figure 2a).

Sirius Red semi‐quantitative analysis showed that the percentage of collagen area in H and OP+ was completely comparable, while collagen staining in OP– zones was reduced compared to H and OP+ (Table 1) (Figure 2b).

**TABLE 1:** Sirius red semi‐quantitative analysis

	Total bone	Cortical bone	Trabecular bone
H	91.77% ± 3.62	93.36% ± 2.85	90.17% ± 3.82
OP+	87.78% ± 4.92	90.17% ± 3.47	84.19% ± 4.9
OP–	72.94% ± 7.37	78.06% ± 2.71	67.82% ± 7.05
ANOVA	*p* < 0.0001	*p* < 0.0001	*p* = 0.0003
Tukey's multiple comparison	H vs. OP+, *p* = 0.174 H vs. OP–, *p* = 0.0001 OP+ vs. OP– *p* = 0.0001	H vs. OP+, *p* = 0.217 H vs. OP–, *p* = 0.0001 OP+ vs. OP– *p* = 0.0004	H vs. OP+, *p* = 0.197 H vs. OP–, *p* = 0.0002 OP+ vs. OP– *p* = 0.004

**FIGURE 3:**
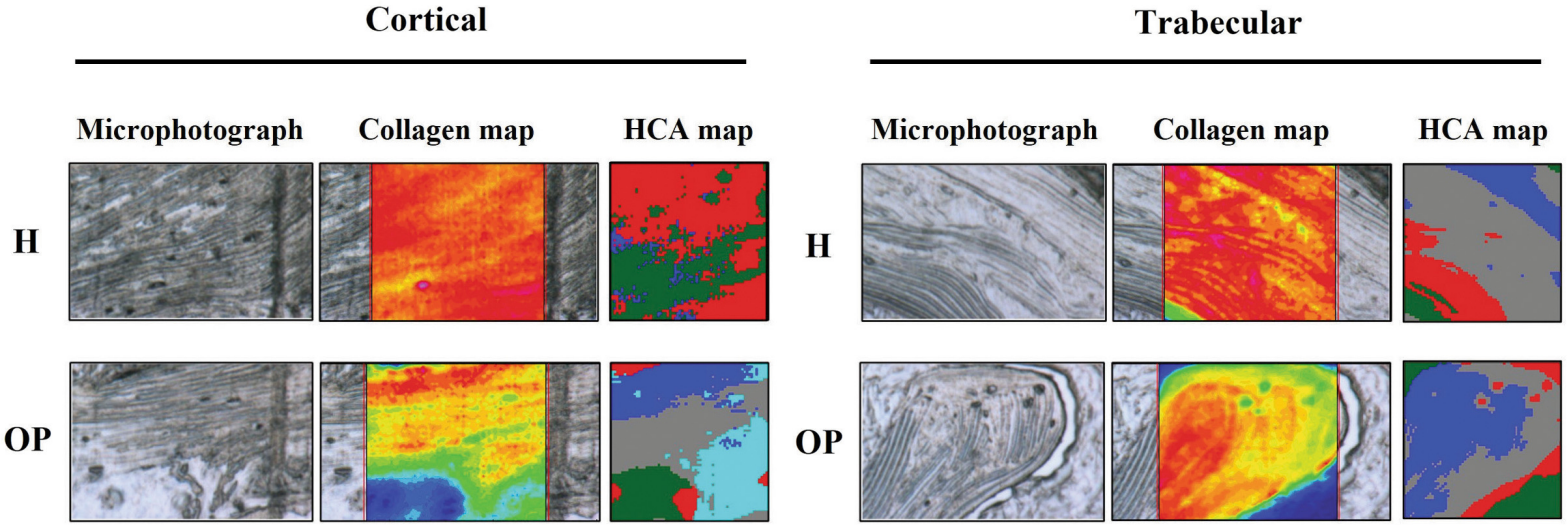
Representative microphotographs of healthy (H) and osteoporotic (OP) samples, in the cortical and trabecular regions; false color images showing the topographical distribution of collagen, and corresponding HCA maps

The hyperspectral imaging analysis of H and OP bone tissues is shown in Figure 3, which displays the microphotographs of representative H and OP samples, the false color images showing the topographical distribution of collagen (Collagen maps), and the corresponding HCA maps. Collagen maps confirmed the results from histological analyses by displaying a homogeneous distribution of collagen within the mapped areas in both H cortical and trabecular bones; conversely, in either OP cortical and trabecular bone samples, a visible general decrease and an inhomogeneous distribution of collagen were found, with areas containing different amounts of this protein. HCA maps also supported these findings, confirming the profile displayed by Collagen maps, both in H and OP cortical and trabecular bone samples.

**FIGURE 4:**
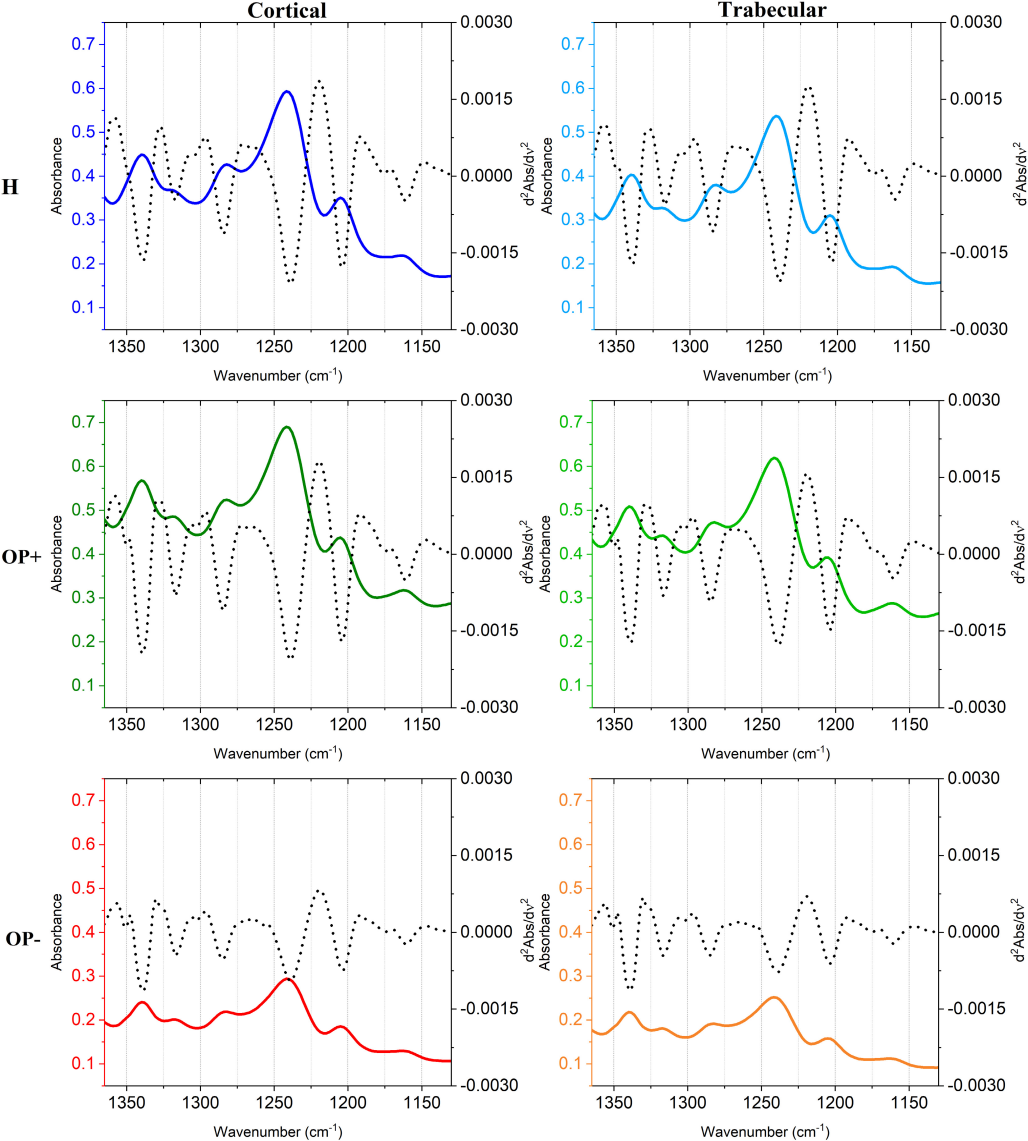
Average spectra of healthy bones (H), collagen‐rich areas of osteoporotic bones (OP+), and collagen‐poor areas of osteoporotic bones (OP–) in cortical and trabecular regions, in the 1360–1130 cm^−1^ spectral range

As regards H samples, collagen‐related spectra were all homogeneous, and, hence, a single average spectrum was calculated from all sections, named H cortical and H trabecular, based on the region of interest. Conversely, in OP osteoporotic samples, two sets of spectra were identified representative of collagen‐rich areas (OP+) and collagen‐poor ones (OP–) (named respectively OP+ cortical, OP+ trabecular, OP– cortical and OP– trabecular, based on the region of interest). The analysis of the average IR spectra, reported in Figure 4 both in absorbance (continuous colored lines) and second derivative mode (dotted black lines), highlighted the following bands: ~1340 cm^−1^ (CH_2_ wagging of proline side chains)^29^, ^30^;~1320 cm^−1^ (a‐helix secondary structures)^31^, ^32^; ~1284 cm^−1^ and ~1240 cm^−1^, (collagen triple helix)^33^; ~1264 cm^−1^ (random secondary structures),^33^ ~1204 cm^−1^ (amino acids lateral chains)^34^ and ~ 1160 cm^−1^ (C‐OH moiety of carbohydrates).^18^, ^35^, ^36^

**FIGURE 5:**
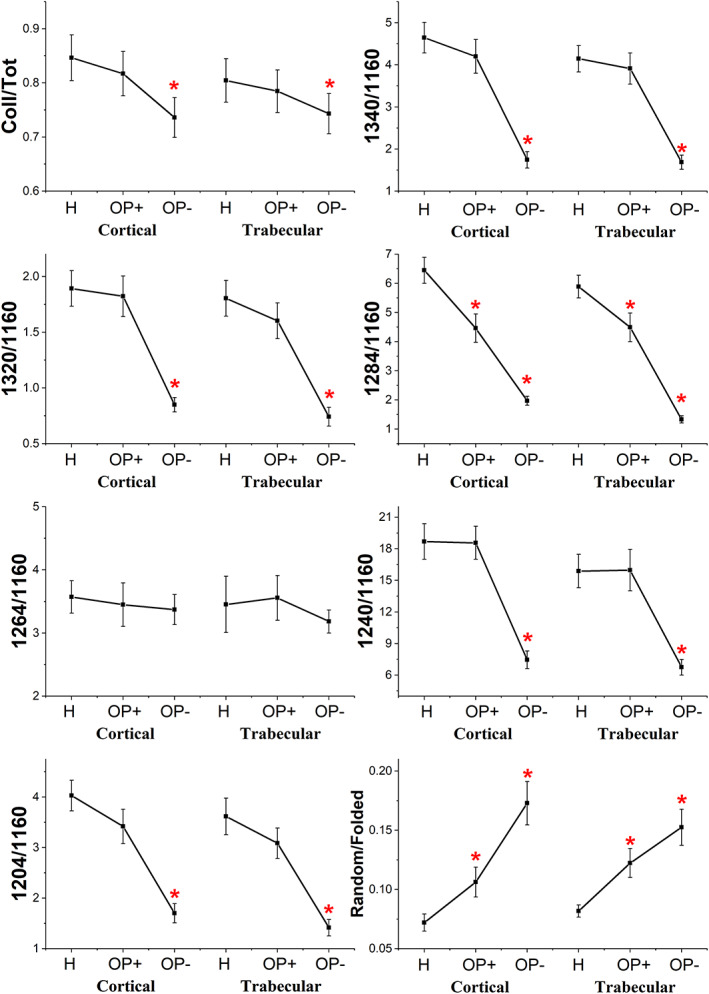
Statistical analysis of the following band area ratios calculated for H, OP+, and OP– spectra in cortical and trabecular regions: Coll/tot, 1340/1160, 1320/1160, 1284/1160, 1264/1160, 1240/1160, 1024/1160, and random/folded. Data are presented as mean ± SD red asterisks indicate statistically significant differences (*p* < 0.05; one‐way ANOVA and Tukey's multiple comparison test)

The following band area ratios diagnostic for collagen composition and structure were calculated and statistically analyzed (Figure 5): Coll/Tot (total collagen), 1340/1160 (collagen's proline), 1320/1160 (a‐helices), 1284/1160 (triple helices), 1264/1160 (random structures), 1240/1160 (triple helices), 1204/1160 (amino acids' lateral chains), and Random/Folded (calculated as the ratio between the areas of the 1264 cm^−1^ band and the sum of the areas of the bands at 1320, 1284^—^, and 1240 cm^−1^). First, it is noteworthy to note that no significant difference was found between cortical and trabecular bones for all the analyzed spectral features. The Coll/Tot band area ratio 1340/1160 and 1204/1160 band area ratios are considered representative of total collagen since they are related respectively to collagen's proline and amino acids' side chains: they significantly decreased in OP– samples with respect to H, while no significant change was observed in OP+, confirming the presence of collagen‐rich and collagen‐poor regions within OP samples. As regards collagen secondary structure, the 1320/1160, 1284/1160, and 1240/1160 band area ratios, attributed to α‐ and triple helices, showed a general decreasing trend in the OP with respect to H. Conversely, the 1264/1160 band area ratio, attributed to random structures, did not change among the samples, while the Random/Folded ratio, calculated to elucidate the random component further, displayed a significant increase both in OP+ and OP– samples. These findings suggest that in collagen‐rich areas of cortical and trabecular OP bones, the collagen component is almost like H one, both in terms of amount and structural organization, whereas in OP– areas, collagen showed a profoundly altered secondary structure characterized by the loss of the typical α‐ and triple helices and abundant random structures.

The statistical analysis of the calculated band area ratios displayed a general condition of decreased collagen content in OP samples, particularly in the OP– spectra. All the band area ratios displayed a decreasing trend: 1340/1160 (collagen's proline), 1320/1160 (a‐helices), 1240/1160 (triple helices), and 1204/1160 (amino acids' lateral chains) showed statistically comparable values for H and OP+ samples, within cortical and trabecular regions, while a significant decrease was found in OP– spectra; as regards 1284/1160 (triple helices) band area ratio, a significant decrease was found in OP+ and, as a further extent, in OP– spectra. Hence, given these results suggest a general decrease in collagen content within some specific areas of OP samples, confirming the histological results. Conversely, the 1264/1160 band area ratio (random structures) displayed no significant change among the three regions, both in the cortical bone and the trabecular one. To further elucidate the misfolded/unfolded component with respect to all the properly folded secondary structures, the 1264/ordered band area ratio was also calculated: a significant increase was found in OP+ and, to a further extent, in OP– spectra.

**FIGURE 6:**
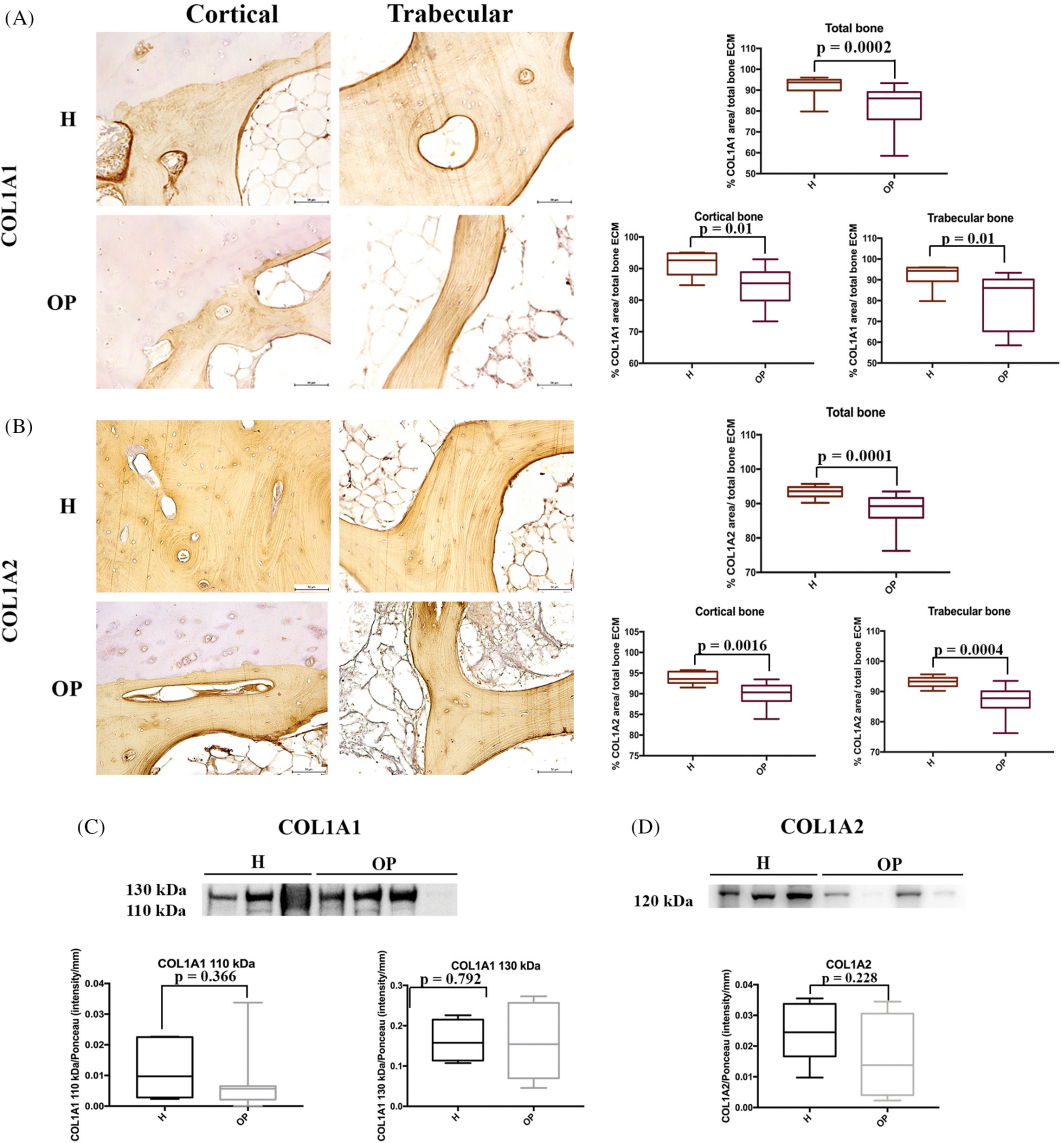
Expression of type I collagen chains (COL1A1 and COL1A2) in healthy (H) and osteoporotic (OP) tissues. (A) Immunohistochemical staining images (×20 magnification, 50 μm scale bar) and semi‐quantification of COL1A1; (B) immunohistochemical staining images (×20 magnification, 50 μm scale bar) and semi‐quantification of COL1A2; (C) representative Western blotting image and densitometry for COL1A1; (D) representative Western blotting image and densitometry for COL1A2

Immunohistochemical analyses were used to analyze the overall expression of Type I Collagen molecule, examining the two chains COL1A1 and COL1A2. Collagen staining was spread throughout the bone ECM with a marked stain along lamellar borders, more evident in H bone. (Figure 6A and B) The semi‐quantitative analysis showed significant differences in both COL1A1 (Figure 6A) and COL1A2 (Figure 6B) staining and content between H and OP samples. (Table 2).

**TABLE 2:** Semi‐quantitative evaluation of histochemical staining

Protein	Total bone ECM			Cortical bone			Trabecular bone		
	Healthy bone	OP bone	*p*‐value	Healthy bone	OP bone	*p*‐value	Healthy bone	OP bone	*p*‐value
COL1A1	91.82% ± 4.80	81.80% ± 10.27	0.0002	91.50% ± 3.94	84.33% ± 6.11	0.01	92.14% ± 6.19	79.57% ± 13.08	0.01
COL1A2	93.23% ± 1.89	88.20% ± 4.17	0.0001	93.30% ± 2.12	89.70% ± 2.65	0.0016	93.16% ± 1.75	86.97% ± 4.86	0.0004
DCN	19.11% ± 6.67	29.47% ± 13.19	0.002	17.55% ± 11.72	30.89% ± 13.5	0.006	20.80% ± 5.58	28.04% ± 13.31	0.235
OCN	14.95% ± 7.86	28.01% ± 12.46	0.0003	16.17% ± 7.50	33.38% ± 14.61	0.01	13.87% ± 8.45	25.57% ± 10.31	0.009
OPN	22.77% ± 5.95	15.94% ± 6.09	0.0002	22.17% ± 5.67	14.19% ± 3.32	0.002	23.75% ± 6.30	17.88% ± 7.89	0.02
BSP‐2	11.86% ± 7.93	10.42% ± 6.58	0.678	14.08% ± 9.07	11.51% ± 7.73	0.651	9.65% ± 3.22	9.32% ± 5.31	0.862
ON	11.12% ± 7.11	10.57% ± 4.18	0.620	14.20% ± 8.16	11.51% ± 3.80	0.798	7.76% ± 5.44	9.45% ± 4.51	0.449
TGF‐β	2.99% ± 1.57	4.71% ± 2.64	0.116	3.68% ± 1.65	4.97% ± 2.05	0.699	2.25% ± 1.16	4.83% ± 3.05	0.222

Abbreviations: COL1A1 and COL1A2, Type I collagen; DCN, Decorin; OCN, Osteocalcin; OPN, Osteopontin; BSP‐2, bone sialoprotein‐2; ON, Osteonectin; TGF‐β, transforming growth factor β.

On the contrary, WB analysis showed no significant differences in the amount of COL1A1 between H and OP bone ECM (*p* = 0.366 for 110 kDa; *p* = 0.792 for 130 kDa) and COL1A2 (*p* = 0.228) (Figure 6C).

#### 3.2.1 Correlations between histological staining and FTIRI data

Pearson's correlations were evaluated between Coll/Tot from FTIRI analysis and area percentage collected from Sirius Red staining and Random/Foiled ratio from FTIRI analysis and area percentage evaluated from Sirius Red staining. We observed positive correlation between Coll/Tot and area percentage in both cortical (r = 0.9982, *p* = 0.019) and trabecular bone (r = 0.9981, *p* = 0.02). Conversely, Random/Foiled and area percentage showed a significant negative correlation in cortical bone (r = −0.9902, *p* = 0.044). This trend was also observed in trabecular bone, despite not being significant (r = −0.9403, *p* = 0.111).

### 3.3 OP tissue exhibited modifications in DCN, OCN, OPN, and BSP‐2 distribution and/or expression

**FIGURE 7:**
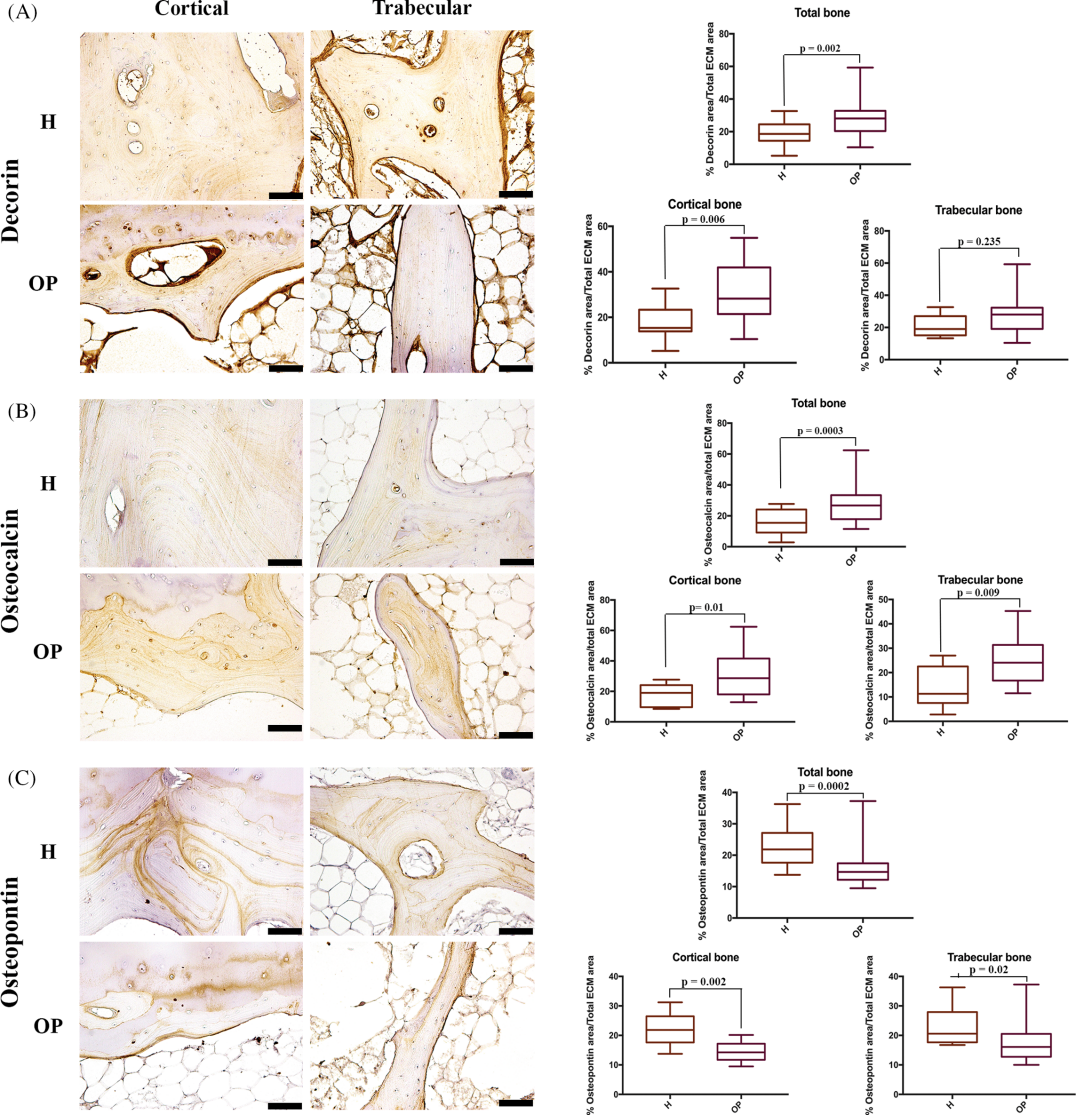
Immunohistochemical staining of (A) Decorin, (B) Osteocalcin, (C) Osteopontin in healthy (H) and osteoporotic (OP) tissues (20x magnification, 50 μm scale bar). (A) Immunohistochemical staining images and semi‐quantification of Decorin; (B) immunohistochemical staining images and semi‐quantification of Osteocalcin; (C) immunohistochemical staining images and semi‐quantification of Osteopontin

DCN staining was distributed along lamellae of the entire bone tissue, with the highest expression in osteons and the outer lamellae of trabecular bone. DCN was significantly more expressed in the cortical bone of OP than in H tissues, whereas in the trabecular bone, the increase was not significantly different (Table 2, Figure 7A).

OCN expression was detected in several bone matrix sites: marked staining was observed at the reversal lines and the bone‐cartilage interface, while a homogenous distribution was shown along the lamellae. OCN expression was higher in lamellae of the deepest regions of the cortical and trabecular bone and weaker in the newest osteons. Greater OCN expression was detected in OP bone samples in comparison to the H ones (Table 2, Figure 7B). Similarly, we observed significantly (*p* = 0.001) higher OCN amount in the serum from OP patients (13.97 μg/ml ± 0.643) than in the H ones (H 6.81 μg/ml ± 0.201).

OPN in the cortical bone accurately demarked the reversal lines and the bone‐articular cartilage interface, with its highest amount observed in the cement lines and several lamellae. The OPN staining was present in the reversal lines and numerous lamellae in trabecular bone. Healthy bone specimens exhibited a higher OPN staining than the OP ones, where a significant increase in both the cortical and trabecular tissues was observed (Table 2, Figure 7C).

**FIGURE 8:**
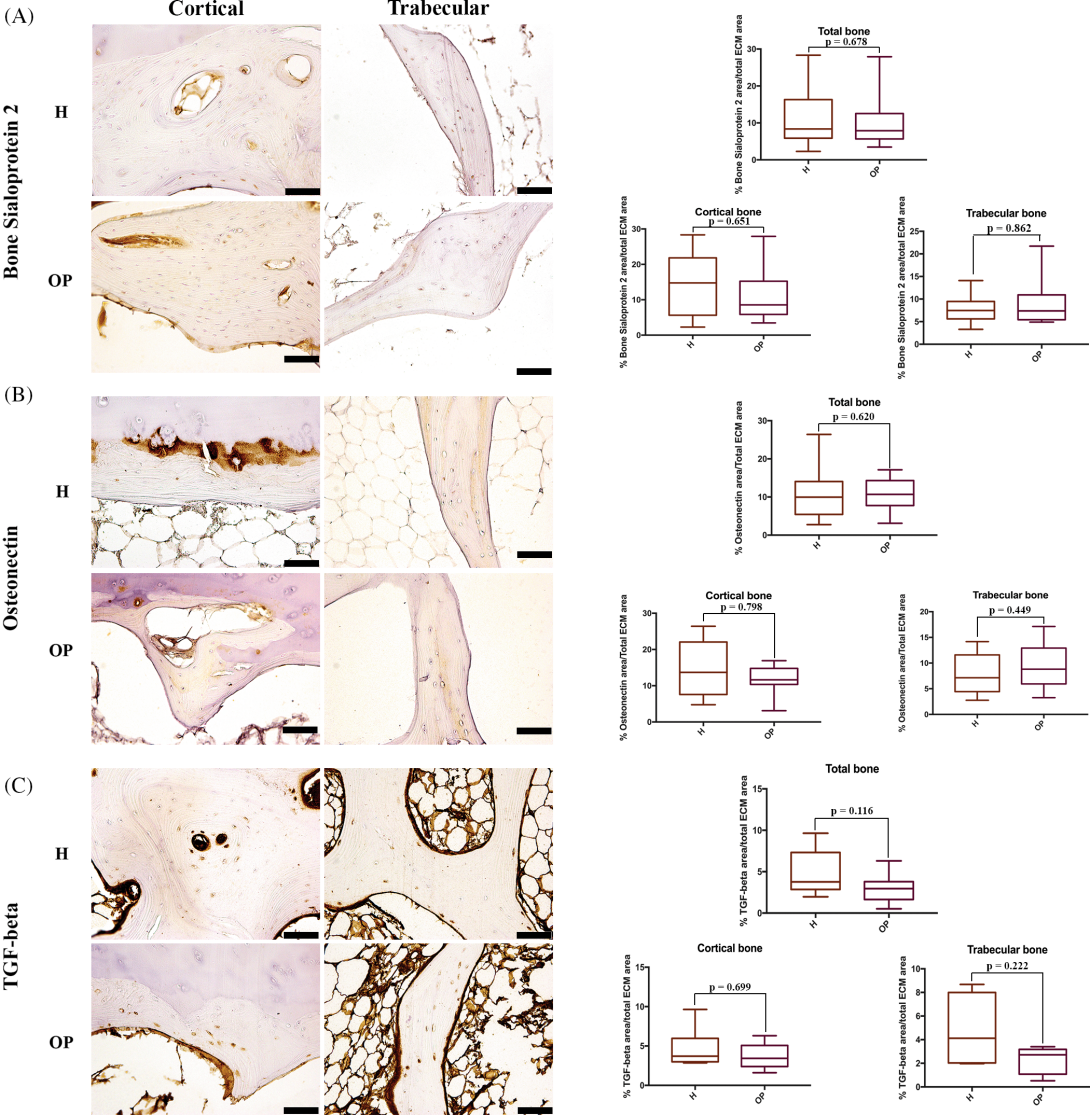
Immunohistochemical staining of (A) bone sialoprotein 2, (B) Osteonectin and (C) TGF‐beta in healthy (H) and osteoporotic (OP) tissues (×20 magnification, 50 μm scale bar). (A) Immunohistochemical staining images and semi‐quantification of bone sialoprotein 2; (B) immunohistochemical staining images and semi‐quantification of Osteonectin; (C) immunohistochemical staining images and semi‐quantification of TGF‐beta

BSP‐2 staining was weak, mainly present in the “young” bone, with the highest expression in the woven bone. In the cortical bone, BSP‐2 positivity was evident in the osteons and the outer lamellae of both cortical and trabecular bone. Moreover, osteocytes expressed a high amount of BSP‐2. No significant differences were found between H and OP specimens (Table 2, Figure 8A).

ON expression was weak, mainly concentrated in the interstitial lamellae of the cortical bone, whereas in the trabecular bone, ON was randomly distributed along lamellae. Higher ON expression was observed in ossified cartilage and woven bone. No significant differences between OP and H bone were detected. Immunohistochemical analysis revealed that in H samples, ON was significantly more expressed in the cortical area than in the trabecular one (*p* = 0.05) (Table 2, Figure 8B).

TGF‐β stain was seen as abundant spots in ECM, as well as in the marrow lacunae. High TGF‐β amount was also expressed in endosteum. Immunostaining analysis revealed no significant differences in TGF‐β stain between OP and H subjects (Table 2, Figure 8C).

**FIGURE 9:**
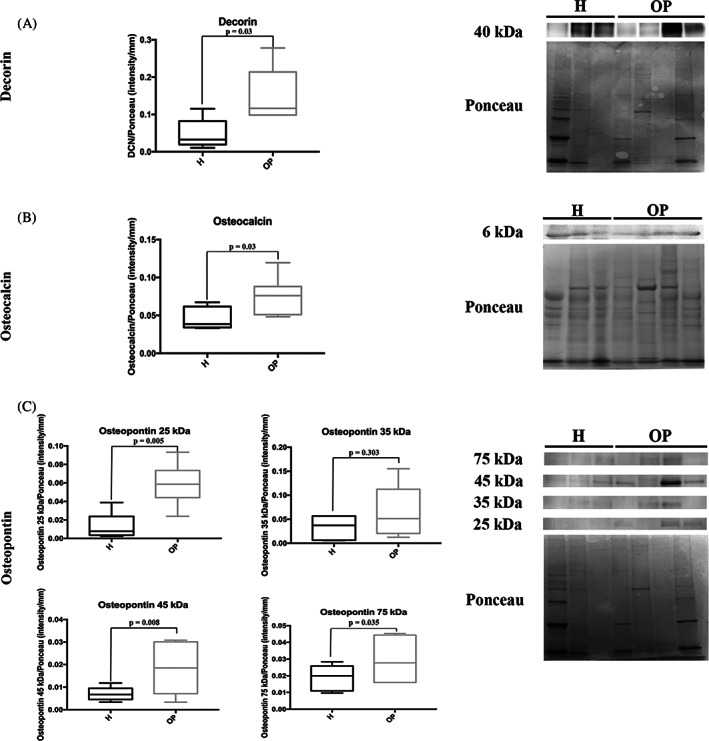
Representative Western blotting image and densitometry for (A) Decorin, (B) Osteocalcin, and (C) Osteopontin in healthy (H) and osteoporotic (OP) tissues (the same Ponceau staining was used to normalize proteins analyzed at different molecular weight on the same membrane)

WB showed an increase of DCN (*p* = 0.03) and OCN (*p* = 0.03) expression in OP bone samples compared to the H ones (Figure 9A and B). By deciphering the amount of the OPN isoforms, we detected a rise in the 25 kDa (*p* = 0.005), 45 kDa (0.008) and 75 kDa (*p* = 0.035) isoforms in OP tissue, while no differences in the 35 kDa protein were noted between the H and OP groups (*p* = 0.303) (Figure 9C).

**FIGURE 10:**
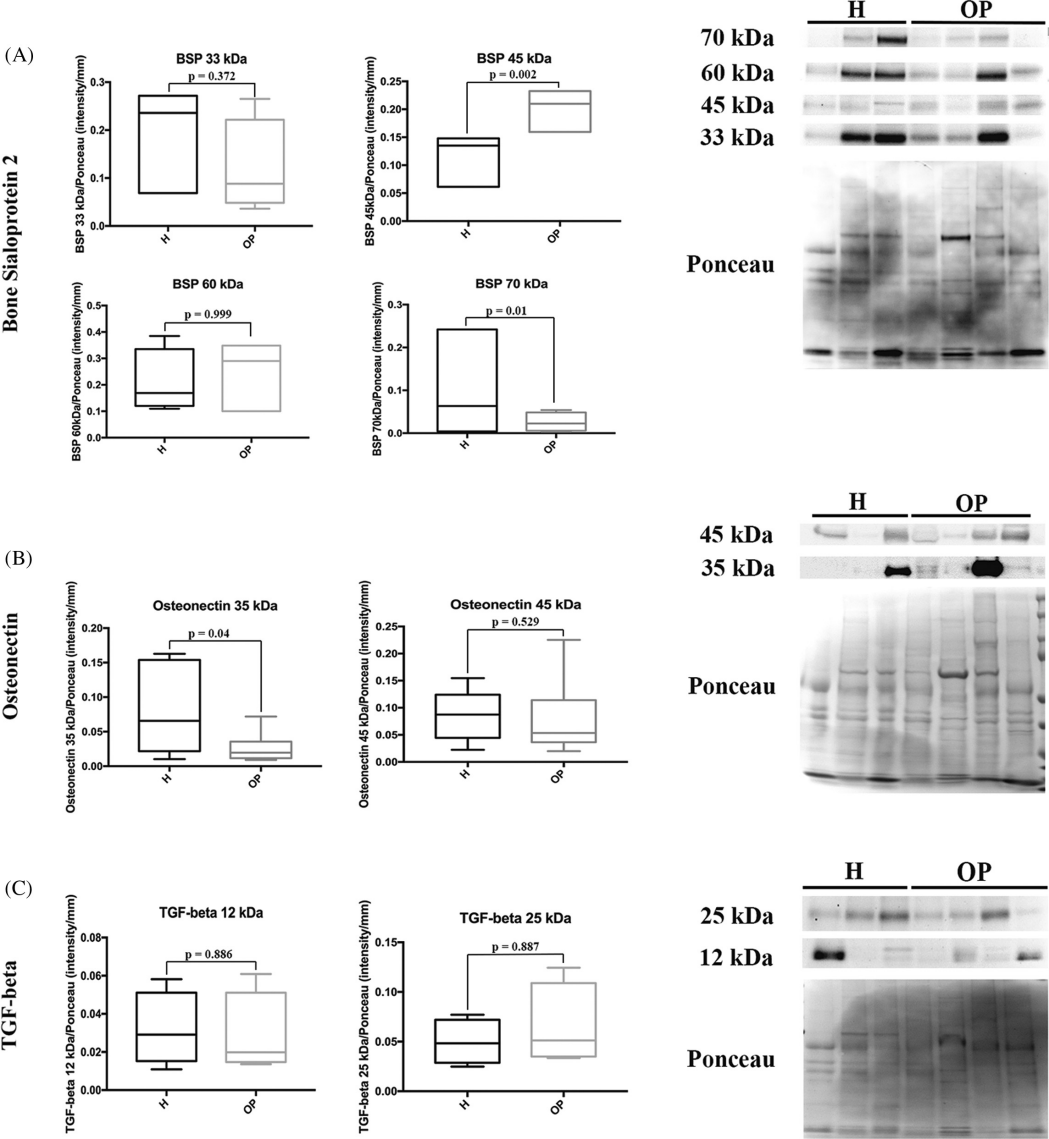
Representative Western blotting image and densitometry for (A) bone sialoprotein 2, (B) Osteonectin, and (C) TGF‐beta in healthy (H) and osteoporotic (OP) tissues (the same Ponceau staining was used to normalize proteins analyzed at different molecular weight on the same membrane)

An increase in the 45 kDa (*p* = 0.002) and 70 kDa (*p* = 0.01) isoforms of BSP‐2 was found in OP tissues in comparison to H bone, while no changes were observed in the 33 kDa (*p* = 0.372) and 60 kDa (*p* = 0.999) BSP‐2 isoforms (Figure 10A). OP bone held less 35 kDa (immature) ON isoform than the H ones (*p* = 0.04), whereas the 45 kDa (mature) protein was equally expressed in both tissues (*p* = 0.529) (Figure 10B). An equal amount of TGF‐β 12 and 25 kDa isoforms were observed in both OP and H tissues (Figure 10C).

#### 3.3.1 Changes in the correlation of protein localization and modification in their expression

Pearson's analysis applied on the different protein staining showed significant correlations between the analyzed molecules in both H and OP bone specimens (Table 3) In H bone samples, negative correlations OPN/DCN and OPN/TGF‐β, BSP‐2/OCN and BSP‐2/ON, COL1A2/ TGF‐β, and TGF‐TGF‐β/DCN were detected while a positive correlation between OCN and ON was observed.

**TABLE 3:** Pearson's correlations in histological staining

H bone							
	**COL1A2**	**DCN**	**OCN**	**OPN**	**BSP‐2**	**ON**	**TGF‐β**
COL1A2	–	0.608	0.407	−0.556	−0.662	0.440	**−0.873**
DCN	0.608	–	−0.478	**−0.998**	0.193	−0.446	**−0.918**
OCN	0.407	−0.478	–	−0.411	**−0.954**	**0.999**	0.090
OPN	−0.556	**−0.998**	−0.411	–	−0.255	0.502	**−0.765**
BSP‐2	−0.662	0.193	**−0.954**	−0.255	–	**−0.964**	0.213
ON	0.440	−0.446	**0.999**	0.502	**−0.964**	–	0.054
TGF‐β	**−0.873**	**−0.918**	0.090	**−0.765**	0.213	0.054	–

OP bone							
	**COL1A2**	**DCN**	**OCN**	**OPN**	**BSP‐2**	**ON**	**TGF‐β**
COL1A2	–	−0.141	−0.501	−0.353	−0.591	−0.189	**0.943**
DCN	−0.141	–	0.108	**0.899**	**0.861**	0.658	0.047
OCN	−0.501	0.108	–	0.533	0.168	−0.527	**−0.989**
OPN	−0.353	**0.899**	0.533	–	**0.814**	0.336	−0.399
BSP‐2	−0.591	**0.861**	0.168	**0.814**	–	**0.749**	−0.330
ON	−0.189	0.658	−0.527	0.336	**0.749**	–	0.250
TGF‐β	**0.943**	0.047	**−0.989**	−0.399	−0.330	0.250	–

*Note*: Bold values are the significant values. Abbreviations: COL1A2, type I collagen; DCN, Decorin; OCN, Osteocalcin; OPN, Osteopontin; BSP‐2, bone sialopreotein‐2; ON, Osteonectin; TGF‐β, transforming growth factor beta.

Our study found differences in correlation trends and values in OP compared to H specimens. We evidenced a positive correlation between COL1A2 and TGF‐ TGF‐β, OPN and DCN, ON and BSP‐2, a negative correlation between OCN and ON, and a loss of correlation between OCN and BSP‐2, DCN and TGF‐β, and OPN and TGF‐β. Moreover, there were positive correlations between BSP‐2 and DCN and OPN and negative correlations between TGF‐β and OCN.

Significant Pearson's correlations were also found between protein isoforms detected in both H and OP bone specimens (Table 1S).

In H bone tissue, significant positive correlations were found between DCN and OCN, COL1A2 and ON and BSP‐2 and OPN isoforms, while negative correlation between DCN and ON and BSP‐2 and OPN isoforms were detected. All these correlations lose significance in OP tissues. Furthermore, we observed that the negative correlations between OCN and 45 and 75 kDa OPN, and 45 kDa ON found in H bone samples reverse the trend in OP specimens, becoming positive (Table 1S).

## 4 DISCUSSION

Altered bone tissue homeostasis affects the balance between matrix composition and mineral content. This leads to subsequent modifications in tissue morphology, matrix arrangement, and disruption of bone architecture.^1^, ^2^, ^4^, ^5^ Currently, histomorphometric analysis of bone in OP subjects^37^, ^38^, ^39^, ^40^, ^41^ is usually performed in certain anatomical region by using micro‐computed tomography (μCT) and/or on undecalcified‐bone sections.^40^, ^41^, ^42^ In this study, a novel multidisciplinary approach combined histological, histomorphometric, molecular, and hyperspectral analyses to investigate morphology, structure, and biochemical composition in H and OP human femoral head. All the methodologies were set and adapted to perform a complete examination of bone tissue in order to obtain further insight into the possible implications of bone ECM changes in the mechanism of OP.

The histological mosaic technique allowed the extensive observation of the femoral head's morphological features by obtaining an entire tissue section reconstruction: OP tissue displayed an increase in resorption sites, fewer and thinner trabeculae, as well as “trabecularization” and reduction in the width of the cortical bone compared to the H tissue. Overall, this indicates an imbalance in bone remodeling at both endosteal (trabecular deletion and cortical loss) and Haversian (cortical trabecularization) surfaces. These observations were corroborated by the histomorphometric analysis, which showed the reduction of the total bone area and cortical and trabecular bone tissue surfaces in the OP samples.^43^, ^44^

The evaluation of bone ECM protein expression and localization is pivotal to understanding bone biology under normal and pathological conditions, as these molecules have multiple effects on bone remodeling (i.e., reciprocal regulation, mediation of cell‐matrix interactions, and influence on cell behaviors).^5^, ^6^, ^45^

Type I Collagen has a central role in bone structural properties and regulates synthesis and function of the other matrix proteins, beyond its pivotal function as the “backbone” for initiation of mineral deposition.^46^ Sirius Red staining and immunohistochemistry showed a homogeneous collagen disposition more marked along the lamellae in H bone, while in OP tissue, the staining was discontinuous, and the area percentage decreased. Focusing our attention on OP samples, we identified zones in which collagen distribution was superimposable to the H bone (OP+) and areas showing decreased and disordered collagen disposition (OP–). These results were also validated by the hyperspectral imaging analysis, which demonstrated different amounts and distribution of collagen within both IR and HCA maps. While H samples displayed a homogeneous pattern of collagen distribution in the cortical and trabecular, within cortical and trabecular OP sections, areas with a collagen content comparable to H samples (OP+) were distinguishable from collagen‐poor areas (OP–). Semi‐quantitative analysis of immunohistochemical staining for COL1A1 and COL1A2 confirmed that the area percentage for both proteins decreased in OP bone compared to H bone, with higher variability in the OP matrix probably due to the differences as mentioned above. In contrast, WB analysis revealed COL1A1 expression was equivalent in H and OP bone tissues, while COL1A2 amount decreased, albeit not significantly, in the pathological tissues. Besides the hyperspectral analysis of IR maps, FTIRI is also used to analyze protein secondary structure state and modification as a biomarker of some pathological conditions.^47^, ^48^, ^49^, ^50^ In fact, by the curve fitting analysis of the Amides I, II, and III convoluted bands, it is possible to unveil the underlying sub‐peaks assigned to specific secondary structures, including triple and α‐helices, β‐sheets, β‐turns, and random coils.^32^, ^51^, ^52^ In particular, the Amide III band, composed of three main peaks centered at ~1284, ~1240, and ~1204 cm^−1^, offers relevant information on the peculiar triple‐stranded helix structure of collagen.^29^, ^33^, ^53^ Specific band area ratios were calculated and employed to investigate the relative amount of collagen and its conformation in terms of secondary structure. The obtained results clearly showed similar values between cortical and trabecular bones, as well as between H and OP+ samples. Conversely, in OP– samples, the spectral modifications induced by osteoporosis were evident: in fact, collagen not only showed a significant decrease (Coll/Tot, 1340/1160, and 1204/1160), but it also displayed a loss of its typical conformation based on α‐ and triple helices (1320/1160, 1284/1160, and 1240/1160), giving way to random, disorganized secondary structures (1264/1160 and Random/Folded).^41^ Further, our results point out on the FTIRI ability to provide an extensive examination of Type I Collagen structure in bone, that could be translate in a future clinical application.^23^

Alterations in molecular composition and mechanical properties of the matrix have consequences on OB behavior. In particular, Type I Collagen structure influences cell adhesion, proliferation, differentiation, and NCPs synthesis, in addition to the matrix mineralization grade.^7^, ^46^, ^54^, ^55^ Altogether, our results lead us to speculate that, in OP bone tissue, Type I Collagen modifications are related to regions (i.e., OP– areas) whereby protein structure and disposition are altered without affecting total collagen amount.^56^ These features can negatively influence not only bone strength but also osteoblast functions and mineralization, affecting several aspects of bone quality.^57^, ^58^

The structure and functions of Type I Collagen are also strictly related to DCN, OCN, ON, BSP‐2 and TGF‐β ECM proteins.^5^ DCN is involved in Type I Collagen fibrils assembly and maturation. This protein also plays a role in the inhibition of the mineralization process as its expression starts to decrease at the onset of matrix mineralization.^5^, ^11^, ^45^ We observed high DCN expression in the osteon and outer lamellae of the trabecular bone, and greater DCN area percentage and amount in OP bone compared to H tissue. An increase in DCN expression denotes the presence of a more disorganized and immature tissue in OP samples, most likely due to a high resorption rate and turnover, which in turn leads to an inadequate maturation of collagen fibers and their incomplete mineralization.^56^

OCN, OPN and Type I Collagen interaction have a synergic effect on bone resorption as well as on mineralization.^16^, ^59^, ^60^, ^61^, ^62^ Bailey et al. showed that only mice knock‐out for both proteins (OCN^−^/OPN^−^) presented increased outer diameter of cortical bone in radii as a consequence of inhibited bone resorption.^62^ OPN is also considered a “glue” at the mineral‐collagen surface since its binding to the collagen matrix supports intrafibrillar mineralization. OPN interaction with Collagen and OCN is considered integral for mature mineralization.^16^, ^63^ In addition, OCN is involved in OCs precursors recruitment at the bone resorption sites and helps their differentiation into mature OCs.^5^, ^6^, ^16^, ^64^ OPN favors the cell‐matrix interactions by binding its RGD sequences with integrins of OCs (ανβ3) and OBs, controlling cell adhesion to the bone surface during remodeling and regulating collagen fibrillogenesis.^45^, ^59^ In our study, we observed that both OCN and OPN were localized at the reversal lines and, while OCN staining increased in OP tissue, OPN staining decreased, as previously shown by Tarquini et al.^65^ We also observed that OCN amount in serum from OP patients was more than 2‐fold compared to that from the H ones. OCN measurement in patient serum is considered a bone resorption marker, as well as C‐terminal (CTX) and N‐terminal (NTX) telopeptides of Type I Collagen detection. High levels of these molecules are indicative for high resorption rate.^1^, ^5^ .

Western Blotting analysis confirmed the increase of OCN expression and in analyzing the four OPN isoforms (25, 35, 45, and 75 kDa), we detected that the 25 kDa (cleavage products), and 45 kDa and 75 kDa ones were more detectable in OP bone. OPN functions are strictly related to the post‐translational modifications, i.e., primarily phosphorylation of the 35 kDa canonical isoform that can assume the 44–75 kDa forms. Phosphorylated OPN exerts a great influence on mineralization and bone‐resorbing by protein‐OCs interaction.^5^, ^66^, ^67^, ^68^, ^69^ The increased expression of both OCN and phosphorylated OPN in OP bone could exert a negative influence on tissue mineralization, as bone resorption may affect the hydroxyapatite (HA) nucleation process induced by both OCN and OPN: the developed acid environment during bone resorption favors the removal of the carboxylate groups from OCN protein, causing loss of OCN‐HA affinity^70^, ^71^, ^72^; the reducing bone laying and increasing bone resorption hamper primary mineralization promoted by OPN during bone formation^63^; and the overexpression of phosphorylated OPN inhibits HA nucleation and favors OCs hyperactivity, with the generation of a higher amount of 25 kDa cleaved products.^68^, ^73^ In H samples, we found a negative correlation between the expression of the 45 and 75 kDa OPN isoforms and OCN. On the contrary, in OP specimens, their positive correlation and localization at the reversal lines could imply the possible cooperation of OPN and OCN in favoring OC recruitment and ECM bonding.

In the bone matrix, BSP‐2 binds fibrillar collagen, and this association is necessary to BSP‐2‐mediated early mineralization.^14^, ^74^ Similar to OPN, the canonical 33 kDa isoform goes up to 70 kDa in bone ECM after post‐translational modifications, and the high grades of glycosylation and sialic acid content in BSP‐2 is fundamental for HA nucleation and enhance osteogenesis.,^71^, ^74^ BSP‐2 mediates cell‐matrix interaction by RGD sequences.^15^ By examining the expression of the different isoforms, we found that the constitutive (33 kDa) and the functionalized (60 kDa) forms were equivalent in both bone tissues, while in OP bone, the levels of the 45 kDa protein increased while the 70 kDa form decreased. These data might indicate a negative consequence on both HA nucleation and osteogenesis in OP bone. Interestingly, we observed a negative correlation between BSP‐2 and OCN staining in H bone, that disappeared in OP tissue. This trend was confirmed comparing the 70 kDa BSP‐2 and OCN. According to BSP‐2 and OCN multivariate functions, we can deduce that their normal antagonist action in osteogenesis, as well as their activity at different times along the bone mineralization process, is lost in OP tissue.

Bone ON, once glycosylated, is capable of binding to Type I Collagen and preventing its degradation. Furthermore, ON favors the interaction between Ca^2+^ and Type I Collagen, thus increasing the local calcium concentration.^5^, ^12^ In H bone, the ON immunohistochemical expression was significantly higher in the cortical than in the trabecular area, as already shown by Derkx et al.,^75^ and this distribution may be attributed to the higher average mineral content of the cortical compared to cancellous bone.^76^ On the contrary, no differences in ON distribution were detected in OP tissue. Moreover, considering the role of glycosylation in ON function,^12^ the lack of differences in the amount of mature glycosylated ON (i.e., 45 kDa isoform) between H and OP tissues, suggested that collagen alterations observed in OP may not be attributable to changes in ON expression.

Finally, TGF‐β, the most present growth factor within bone ECM, exerts an important role in the regulation of some ECM proteins (i.e., Type I Collagen, ON and OPN) and in controlling mineralization.^5^ Data from the literature on a possible correlation between TGF‐β and OP onset and/or maintenance are controversial.^77^, ^78^, ^79^ Neither immunohistochemistry nor WB analysis showed any difference in TGF‐β expression between H and OP bone tissues. The immunohistochemical staining was detected as dots representing the stored and inactive protein in all analyzed samples.^80^ This data compounds the hypothesis that the TGF‐β involvement in OP development may not be related to the amount of protein stored in the bone ECM but to the active circulating form released during bone resorption and/or different polymorphisms.^79^, ^81^

There are a few constraints to our study. First, the limited number of bone samples that is due to the sparse availability of femoral heads after hip arthroplasty, especially from healthy subjects. Another weakness could be represented by the small number (eight) of proteins investigated considering the approximately 200 NCPs in bone. Indeed, prior to this study, we performed in‐depth bibliographic research for deciphering the main proteins that could be involved in OP onset and maintenance.

## 5 CONCLUSIONS

Overall, our novel multidisciplinary approach has confirmed bone architecture changes and alterations of bone area fraction cortical and trabecular thickness in OP tissue. Furthermore, we have shown that OP bone contains several zones with deep Type I Collagen structural defects, affecting bone strength and structure. These collagen modifications, which are associated with changes in certain NCPs expressions (e.g., OCN, OPN, and BSP‐2), suggest a crucial role of these ECM proteins in OP pathophysiology. The ECM proteins' role may be characterized by changes in their cooperative or antagonist role in normal bone biology. Although our work does not have a direct clinical application, we attempt to provide an exhaustive evaluation of the intricate bone ECM network and its role on bone homeostasis, which is still not fully addressed in the literature. This knowledge could be crucial for designing targeted clinical strategies to preserve bone mass in OP and developing a new therapeutic approach in bone diseases. In addition, our study highlights the FTIRI potential in Type I Collagen analysis in bone, strengthening its translational capability in future support for traditional diagnostic methods.

## CONFLICT OF INTEREST

None declared.

## Supporting information


**Appendix S1** Supporting Information

## Data Availability

The data that support the findings of this study are available from the corresponding author upon reasonable request.
