# Supplementary material for: Altered type I collagen networking in osteoporotic human femoral head revealed by histomorphometric and Fourier transform infrared imaging correlated analyses
Source: Biofactors. 2022 Jun 6;48(5):1089–110. doi: 10.1002/biof.1870 (PMC9796100; doi:10.1002/biof.1870)

**­Supplementary material**

Table 1S- Pearson's correlations in protein expression

Table 2S- Antibodies used in immunohistochemistry (IHC) and Western Blotting (WB)

Figure 1S- Triplicates of Western Blot for Col1a1, Col1a2 and Decorin (the same Ponceau staining was used to normalize proteins analyzed on the same membrane at different molecular weights)

Figure 2S- Triplicates of Western Blot for Osteocalcin, Osteopontin and Bone Sialoprotein 2 (the same Ponceau staining was used to normalize proteins analyzed on the same membrane at different molecular weights)

Figure 3S- Triplicates of Western Blot for Osteonectin and Transforming Growth Factor beta1 (the same Ponceau staining was used to normalize proteins analyzed on the same membrane at different molecular weights)

|  | Table 1S- Pearson's correlations in protein expression | | | | | | | | | | | | | | | |  |
| --- | --- | --- | --- | --- | --- | --- | --- | --- | --- | --- | --- | --- | --- | --- | --- | --- | --- |
| H | | | | | | | | | | | | | | | | | |
|  | **COL1A1** | **COL1A2** | **DCN** | **OCN** | **OPN 35** | **OPN 45** | **OPN 60** | **OPN 70** | **BSP-2 33** | **BSP-2 45** | **BSP-2 60** | **BSP-2 70** | **ON 35** | **ON 45** | **TGF-BETA 12** | **TGF-BETA 25** |  |
| COL1A1 |  |  | -0,6217 | 0,1225 | -0,9968 | 0,7715 | -0,09451 | 0,5928 | 0,9917 | -0,3584 | 0,9976 | 0,4525 | 0,579 | 0,6241 | -0,977 | 0,6841 |  |
| COL1A2 |  |  | -0,966 | -0,4931 | -0,848334 | 0,99869 | 0,517417 | 0,956006 | 0,872860 | -0,84419 | 0,841956 | 0,894846 | 0,966805 | 0,957988 | -0,37565 | 0,738342 |  |
| DCN | -0,6217 | -0,966 |  | 0,701175 | 0,682725 | -0,97798 | -0,72095 | -0,99933 | -0,71713 | 0,95403 | -0,67395 | -0,97979 | -0,99999 | -0,99956 | 0,926556 | -0,50665 |  |
| OCN | 0,1225 | -0,4931 | 0,701175 |  | -0,04225 | -0,537 | -0,99960 | -0,7266 | -0,00593 | 0,882629 | 0,054171 | -0,82961 | -0,69905 | -0,72193 | 0,092180 | -0,64012 |  |
| OPN 35 | -0,9968 | -0,848334 | 0,682725 | -0,04225 |  |  |  |  | -0,99883 | 0,432355 | -0,99992 | -0,52279 | -0,68489 | -0,66084 | 0,990958 | -0,7405 |  |
| OPN 45 | 0,7715 | 0,99869 | -0,97798 | -0,537 |  |  |  |  | 0,846772 | -0,87049 | 0,813271 | 0,91649 | 0,978604 | 0,971397 | -0,8895 | 0,991842 |  |
| OPN 60 | -0,09451 | 0,517417 | -0,72095 | -0,9996 |  |  |  |  | 0,034060 | -0,8955 | -0,02606 | 0,844986 | 0,718891 | 0,741110 | -0,12015 | 0,661479 |  |
| OPN 70 | 0,5928 | 0,956006 | -0,99933 | -0,7266 |  |  |  |  | 0,691316 | -0,96430 | 0,646642 | 0,98642 | 0,999225 | 0,999976 | -0,75107 | 0,99295 |  |
| BSP-2 33 | 0,9917 | 0,87286 | -0,71713 | -0,00593 | -0,99883 | 0,846772 | 0,03406 | 0,691316 |  |  |  |  | 0,719204 | 0,696236 | -0,9962 | 0,77205 |  |
| BSP-2 45 | -0,3584 | -0,84419 | 0,95403 | 0,882629 | 0,432355 | -0,87049 | -0,8955 | -0,96430 |  |  |  |  | -0,95313 | -0,96247 | 0,549429 | -0,92613 |  |
| BSP-2 60 | 0,9976 | 0,841956 | -0,67395 | 0,054171 | -0,99992 | 0,813271 | -0,02606 | 0,646642 |  |  |  |  | 0,676148 | 0,65183 | -0,98928 | 0,732467 |  |
| BSP-2 70 | 0,4525 | 0,894846 | -0,97979 | -0,82961 | -0,52279 | 0,91649 | 0,844986 | 0,98642 |  |  |  |  | 0,979196 | 0,98527 | -0,63243 | 0,960012 |  |
| ON 35 | 0,579 | 0,966806 | -0,99999 | -0,69905 | -0,68489 | 0,978604 | 0,718891 | 0,999225 | 0,719204 | -0,95313 | 0,676148 | 0,979196 |  |  | -0,77646 | 0,996848 |  |
| ON 45 | 0,6241 | 0,957988 | -0,99956 | -0,72193 | -0,66084 | 0,971397 | 0,741110 | 0,999976 | 0,696236 | -0,96247 | 0,65183 | 0,985275 |  |  | -0,75556 | 0,993742 |  |
| TGF-BETA 12 | -0,977 | -0,37565 | 0,926556 | 0,09218 | 0,990958 | -0,8895 | -0,12015 | -0,75107 | -0,9962 | 0,549429 | -0,98928 | -0,63243 | -0,77646 | -0,75556 |  |  |  |
| TGF-BETA 25 | 0,6841 | 0,738342 | -0,50665 | -0,64012 | -0,740538 | 0,991842 | 0,661479 | 0,99295 | 0,77205 | -0,92613 | 0,732467 | 0,960012 | 0,996848 | 0,993742 |  |  |  |
| OP | | | | | | | | | | | | | | | | | |
|  | **COL1A1** | **COL1A2** | **DCN** | **OCN** | **OPN 35** | **OPN 45** | **OPN 60** | **OPN 70** | **BSP-2 33** | **BSP-2 45** | **BSP-2 60** | **BSP-2 70** | **ON 35** | **ON 45** | **TGF-BETA 12** | **TGF-BETA 25** |  |
| COL1A1 |  |  | -0,5881 | 0,01426 | -0,3779 | 0,8238 | -0,3762 | -0,1327 | 0,6252 | -0,3155 | 0,3249 | 0,8914 | 0,579 | 0,5983 | -0,8028 | 0,64 |  |
| COL1A2 |  |  | -0,6541 | 0,2647 | 0,582632 | 0,386814 | 0,427284 | 0,168038 | 0,790280 | 0,554748 | 0,862688 | 0,505144 | 0,659432 | 0,558917 | -0,9117 | 0,984029 |  |
| DCN | -0,5881 | -0,6541 |  | 0,393719 | 0,231404 | -0,34503 | 0,392039 | 0,52767 | -0,571104 | 0,254156 | -0,39106 | -0,58639 | -0,33783 | -0,02895 | 0,774588 | -0,99660 |  |
| OCN | 0,01426 | 0,2647 | 0,393719 |  | 0,8033891 | 0,566 | 0,882277 | 0,9864 | 0,524831 | 0,857635 | 0,673622 | 0,36195 | 0,717219 | 0,906095 | 0,441115 | 0,580821 |  |
| OPN 35 | -0,3779 | 0,582632 | 0,231404 | 0,8033891 |  |  |  |  | 0,444912 | 0,995159 | 0,719524 | 0,075709 | 0,536047 | 0,734839 | 0,494553 | 0,451824 |  |
| OPN 45 | 0,8238 | 0,386815 | -0,34503 | 0,566 |  |  |  |  | 0,870674 | 0,265239 | 0,718032 | 0,962286 | 0,91787 | 0,801636 | -0,4565 | 0,905240 |  |
| OPN 60 | -0,3762 | 0,427284 | 0,392039 | 0,88227718 |  |  |  |  | 0,37825 | 0,989253 | 0,649091 | 0,051320 | 0,51843 | 0,753675 | 0,600766 | 0,402981 |  |
| OPN 70 | -0,1327 | 0,168038 | 0,527670 | 0,9864 |  |  |  |  | 0,383476 | 0,864588 | 0,568472 | 0,205458 | 0,594032 | 0,826686 | 0,582726 | 0,442014 |  |
| BSP-2 33 | 0,6252 | 0,790281 | -0,57110 | 0,524831 | 0,444912 | 0,870674 | 0,37825 | 0,383476 |  |  |  |  | 0,963537 | 0,835608 | -0,49568 | 0,995716 |  |
| BSP-2 45 | -0,3155 | 0,554749 | 0,254156 | 0,857635 | 0,995159 | 0,265239 | 0,989253 | 0,864588 |  |  |  |  | 0,58823 | 0,787801 | 0,489611 | 0,494494 |  |
| BSP-2 60 | 0,3249 | 0,862688 | -0,39106 | 0,673622 | 0,719524 | 0,718032 | 0,649091 | 0,568472 |  |  |  |  | 0,931563 | 0,899583 | -0,2175 | 0,933568 |  |
| BSP-2 70 | 0,8914 | 0,505144 | -0,58639 | 0,361950 | 0,075709 | 0,962286 | 0,05132 | 0,205458 |  |  |  |  | 0,88 | 0,687223 | -0,6696 | 0,918438 |  |
| ON 35 | 0,579 | 0,659432 | -0,33783 | 0,717219 | 0,536047 | 0,917876 | 0,51843 | 0,594032 | 0,963537 | 0,588230 | 0,931563 | 0,88 |  |  | -0,77646 | 0,982581 |  |
| ON 45 | 0,5983 | 0,558917 | -0,02895 | 0,906095 | 0,734839 | 0,801636 | 0,753675 | 0,826686 | 0,835608 | 0,787801 | 0,899583 | 0,687223 |  |  | -0,75556 | 0,870097 |  |
| TGF-BETA 12 | -0,8028 | -0,91170 | 0,774588 | 0,441115 | 0,494553 | -0,4565 | 0,600766 | 0,582726 | -0,49568 | 0,489611 | -0,2175 | -0,6696 | -0,77646 | -0,75556 |  |  |  |
| TGF-BETA 25 | 0,64 | 0,984029 | -0,99660 | 0,580821 | 0,451824 | 0,905240 | 0,402981 | 0,442014 | 0,995716 | 0,494494 | 0,933568 | 0,918438 | 0,982581 | 0,870097 |  |  |  |

|  | **Table 2S- Pearson's correlations in protein expression** | | | | | | | | | | | | | | | |
| --- | --- | --- | --- | --- | --- | --- | --- | --- | --- | --- | --- | --- | --- | --- | --- | --- |
|  | **H** | | | | | | | | | | | | | | | |
|  | **COL1A1** | **COL1A2** | **DCN** | **OCN** | **OPN 35** | **OPN 45** | **OPN 60** | **OPN 70** | **BSP-2 33** | **BSP-2 45** | **BSP-2 60** | **BSP-2 70** | **ON 35** | **ON 45** | **TGF-BETA 12** | **TGF-BETA 25** |
| **COL1A1** |  |  | -0,6217 | 0,1225 | -0,9968 | 0,7715 | -0,09451 | 0,5928 | 0,9917 | -0,3584 | 0,9976 | 0,4525 | 0,579 | 0,6241 | -0,977 | 0,6841 |
| **COL1A2** |  |  | -0,966 | -0,4931 | -0,848334 | 0,99869235 | 0,51741723 | 0,95600647 | 0,87286031 | -0,8441987 | 0,84195628 | 0,89484621 | 0,96680598 | 0,95798814 | -0,3756551 | 0,73834214 |
| **DCN** | -0,6217 | -0,966 |  | 0,70117514 | 0,68272515 | -0,9779893 | -0,7209519 | -0,9993383 | -0,7171391 | 0,9540321 | -0,6739584 | -0,9797945 | -0,9999956 | -0,9995635 | 0,92655602 | -0,5066547 |
| **OCN** | 0,1225 | -0,4931 | 0,70117514 |  | -0,0422536 | -0,537 | -0,9996044 | -0,7266 | -0,0059367 | 0,88262965 | 0,05417147 | -0,8296103 | -0,6990558 | -0,7219334 | 0,09218053 | -0,6401239 |
| **OPN 35** | -0,9968 | -0,848334 | 0,68272515 | -0,0422536 |  |  |  |  | -0,9988385 | 0,43235508 | -0,9999288 | -0,5227904 | -0,6848909 | -0,6608403 | 0,99095807 | -0,740538 |
| **OPN 45** | 0,7715 | 0,99869235 | -0,9779893 | -0,537 |  |  |  |  | 0,84677227 | -0,8704984 | 0,81327193 | 0,9164962 | 0,97860433 | 0,97139799 | -0,889506 | 0,99184283 |
| **OPN 60** | -0,09451 | 0,51741723 | -0,7209519 | -0,9996044 |  |  |  |  | 0,03406066 | -0,895502 | -0,0260646 | 0,84498642 | 0,71889188 | 0,74111055 | -0,1201512 | 0,66147977 |
| **OPN 70** | 0,5928 | 0,95600647 | -0,9993383 | -0,7266 |  |  |  |  | 0,69131621 | -0,9643016 | 0,64664228 | 0,98642077 | 0,99922598 | 0,99997667 | -0,7510718 | 0,9929558 |
| **BSP-2 33** | 0,9917 | 0,87286031 | -0,7171391 | -0,0059367 | -0,9988385 | 0,84677227 | 0,03406066 | 0,69131621 |  |  |  |  | 0,71920451 | 0,69623618 | -0,996272 | 0,7720583 |
| **BSP-2 45** | -0,3584 | -0,8441987 | 0,9540321 | 0,88262965 | 0,43235508 | -0,8704984 | -0,895502 | -0,9643016 |  |  |  |  | -0,9531383 | -0,96247 | 0,54942903 | -0,9261331 |
| **BSP-2 60** | 0,9976 | 0,84195628 | -0,6739584 | 0,05417147 | -0,9999288 | 0,81327193 | -0,0260646 | 0,64664228 |  |  |  |  | 0,67614814 | 0,6518382 | -0,9892866 | 0,73246703 |
| **BSP-2 70** | 0,4525 | 0,89484621 | -0,9797945 | -0,8296103 | -0,5227904 | 0,9164962 | 0,84498642 | 0,98642077 |  |  |  |  | 0,97919657 | 0,98527577 | -0,6324397 | 0,96001246 |
| **ON 35** | 0,579 | 0,96680598 | -0,9999956 | -0,6990558 | -0,6848909 | 0,97860433 | 0,71889188 | 0,99922598 | 0,71920451 | -0,9531383 | 0,67614814 | 0,97919657 |  |  | -0,7764619 | 0,99684815 |
| **ON 45** | 0,6241 | 0,95798814 | -0,9995635 | -0,7219334 | -0,6608403 | 0,97139799 | 0,74111055 | 0,99997667 | 0,69623618 | -0,96247 | 0,6518382 | 0,98527577 |  |  | -0,7555646 | 0,99374206 |
| **TGF-BETA 12** | -0,977 | -0,3756551 | 0,92655602 | 0,09218053 | 0,99095807 | -0,889506 | -0,1201512 | -0,7510718 | -0,996272 | 0,54942903 | -0,9892866 | -0,6324397 | -0,7764619 | -0,7555646 |  |  |
| **TGF-BETA 25** | 0,6841 | 0,73834214 | -0,5066547 | -0,6401239 | -0,740538 | 0,99184283 | 0,66147977 | 0,9929558 | 0,7720583 | -0,9261331 | 0,73246703 | 0,96001246 | 0,99684815 | 0,99374206 |  |  |
|  | **OP** | | | | | | | | | | | | | | | |
|  | **COL1A1** | **COL1A2** | **DCN** | **OCN** | **OPN 35** | **OPN 45** | **OPN 60** | **OPN 70** | **BSP-2 33** | **BSP-2 45** | **BSP-2 60** | **BSP-2 70** | **ON 35** | **ON 45** | **TGF-BETA 12** | **TGF-BETA 25** |
| **COL1A1** |  |  | -0,5881 | 0,01426 | -0,3779 | 0,8238 | -0,3762 | -0,1327 | 0,6252 | -0,3155 | 0,3249 | 0,8914 | 0,579 | 0,5983 | -0,8028 | 0,64 |
| **COL1A2** |  |  | -0,6541 | 0,2647 | 0,58263272 | 0,38681468 | 0,42728434 | 0,16803817 | 0,79028062 | 0,55474872 | 0,86268837 | 0,50514453 | 0,65943259 | 0,55891715 | -0,9117023 | 0,98402932 |
| **DCN** | -0,5881 | -0,6541 |  | 0,39371993 | 0,23140449 | -0,3450381 | 0,39203919 | 0,52767088 | -0,571104 | 0,25415626 | -0,3910653 | -0,5863922 | -0,3378389 | -0,0289533 | 0,77458809 | -0,9966083 |
| **OCN** | 0,01426 | 0,2647 | 0,39371993 |  | 0,8033891 | 0,566 | 0,88227718 | 0,9864 | 0,52483161 | 0,85763569 | 0,67362262 | 0,36195032 | 0,71721958 | 0,90609544 | 0,44111579 | 0,58082151 |
| **OPN 35** | -0,3779 | 0,58263272 | 0,23140449 | 0,8033891 |  |  |  |  | 0,44491275 | 0,99515913 | 0,71952412 | 0,07570915 | 0,53604777 | 0,73483934 | 0,49455358 | 0,45182492 |
| **OPN 45** | 0,8238 | 0,38681468 | -0,3450381 | 0,566 |  |  |  |  | 0,87067494 | 0,26523916 | 0,71803257 | 0,96228654 | 0,9178761 | 0,80163625 | -0,456586 | 0,90524025 |
| **OPN 60** | -0,3762 | 0,42728434 | 0,39203919 | 0,88227718 |  |  |  |  | 0,3782507 | 0,98925385 | 0,64909166 | 0,05132045 | 0,51843027 | 0,75367561 | 0,60076602 | 0,40298104 |
| **OPN 70** | -0,1327 | 0,16803817 | 0,52767088 | 0,9864 |  |  |  |  | 0,38347626 | 0,86458884 | 0,56847258 | 0,20545808 | 0,59403206 | 0,82668695 | 0,58272629 | 0,44201465 |
| **BSP-2 33** | 0,6252 | 0,79028062 | -0,571104 | 0,52483161 | 0,44491275 | 0,87067494 | 0,3782507 | 0,38347626 |  |  |  |  | 0,96353769 | 0,83560809 | -0,4956822 | 0,99571694 |
| **BSP-2 45** | -0,3155 | 0,55474872 | 0,25415626 | 0,85763569 | 0,99515913 | 0,26523916 | 0,98925385 | 0,86458884 |  |  |  |  | 0,58823037 | 0,78780166 | 0,48961172 | 0,49449425 |
| **BSP-2 60** | 0,3249 | 0,86268837 | -0,3910653 | 0,67362262 | 0,71952412 | 0,71803257 | 0,64909166 | 0,56847258 |  |  |  |  | 0,93156336 | 0,89958336 | -0,2175011 | 0,93356842 |
| **BSP-2 70** | 0,8914 | 0,50514453 | -0,5863922 | 0,36195032 | 0,07570915 | 0,96228654 | 0,05132045 | 0,20545808 |  |  |  |  | 0,88000035 | 0,68722374 | -0,6696067 | 0,91843896 |
| **ON 35** | 0,579 | 0,65943259 | -0,3378389 | 0,71721958 | 0,53604777 | 0,9178761 | 0,51843027 | 0,59403206 | 0,9635377 | 0,58823037 | 0,93156336 | 0,88000035 |  |  | -0,7764619 | 0,98258196 |
| **ON 45** | 0,5983 | 0,55891715 | -0,0289533 | 0,90609544 | 0,73483934 | 0,80163625 | 0,75367561 | 0,82668695 | 0,83560809 | 0,78780166 | 0,89958336 | 0,68722374 |  |  | -0,7555646 | 0,87009737 |
| **TGF-BETA 12** | -0,8028 | -0,9117023 | 0,77458809 | 0,44111579 | 0,49455358 | -0,456586 | 0,60076602 | 0,58272629 | -0,4956822 | 0,48961172 | -0,2175011 | -0,6696067 | -0,7764619 | -0,7555646 |  |  |
| **TGF-BETA 25** | 0,64 | 0,98402932 | -0,9966083 | 0,58082151 | 0,45182492 | 0,90524025 | 0,40298104 | 0,44201465 | 0,99571694 | 0,49449425 | 0,93356842 | 0,91843896 | 0,98258196 | 0,87009737 |  |  |

| Table 2S- Antibodies used in immunohistochemistry (IHC) and Western Blotting (WB) | | | | | |
| --- | --- | --- | --- | --- | --- |
| **REAGENT** | **ANTIBODY** | **DILUTION** | **CATALOG** | **SOURCE** | **RRID** |
| ***Human Col1a1*** | Mouse monoclonal | 1:1000 (IHC)  1:500 (WB) | C2456 | Sigma-Aldrich, St. Louis, MO, USA | AB_476836 |
| ***Human Col1a2*** | Rabbit polyclonal | 1:200 (IHC)  1:1000 (WB) | 14695-1-AP | Proteintech, Manchester, UK | AB_2082037 |
| ***Human Decorin*** | Rabbit polyclonal | 1:400 (IHC)  1:1000 (WB) | Ab-175404 | Abcam, Cambridge, UK | AB_2890261 |
| ***Human Osteocalcin*** | Mouse monoclonal | 1:50 (IHC)  1:200 (WB) | SC-365797 | Santa Cruz Biotechnology, Dallas, TX, USA | AB_10859392 |
| ***Human Osteopontin*** | Mouse monoclonal | 1:250 (IHC)  1:200 (WB) | SC-73631 | Santa Cruz Biotechnology | AB_2194995 |
| ***Human Transforming Growth Factor- β*** | Mouse monoclonal | 1:200 (IHC)  1:2000 (WB) | GTX21279 | Genetex, Irvine, CA, USA | AB_373225 |
| ***Human Bone Sialoprotein- 2*** | Mouse monoclonal | 1:100 (IHC)  1:200 (WB) | SC-73630 | Santa Cruz Biotechnology | AB_1119720 |
| ***Envision Dako REAL™ EnVision™ Detection System*** | Anti-mouse, anti-rabbit | N/A | K5007 | Dako, Santa Clara, CA, USA | AB_2888627 |
| ***Secondary antibody conjugated with horseradish peroxidase*** | Anti-mouse | 1:15000 | A190-116P | Bethyl Laboratories, Montgomery, TX, USA | AB_67316 |
| ***Secondary antibody conjugated with horseradish peroxidase*** | Anti-rabbit | 1:5000 | SC-2004 | Santa Cruz Biotechnology | AB_631746 |

Figure 1S


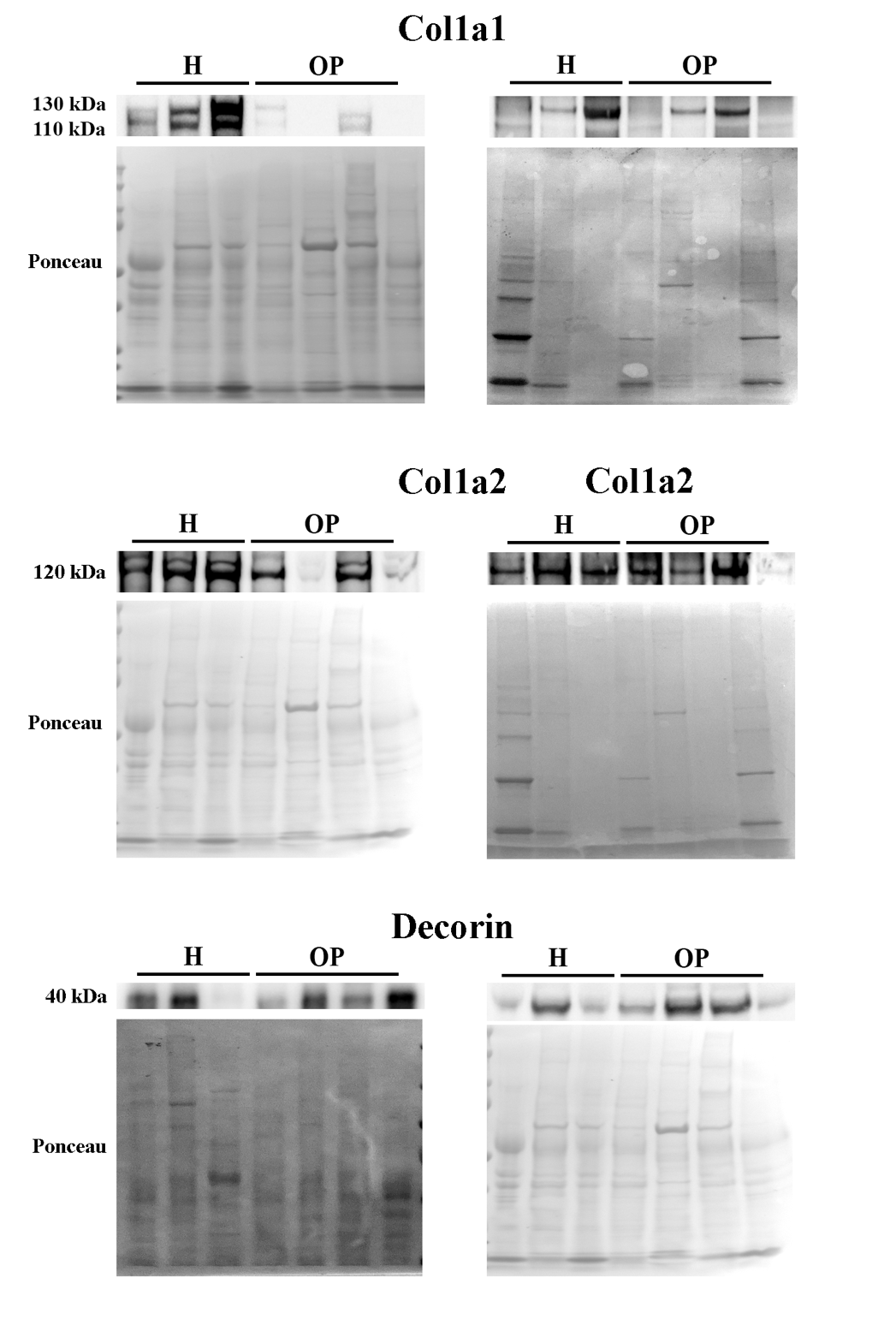


Figure 2S


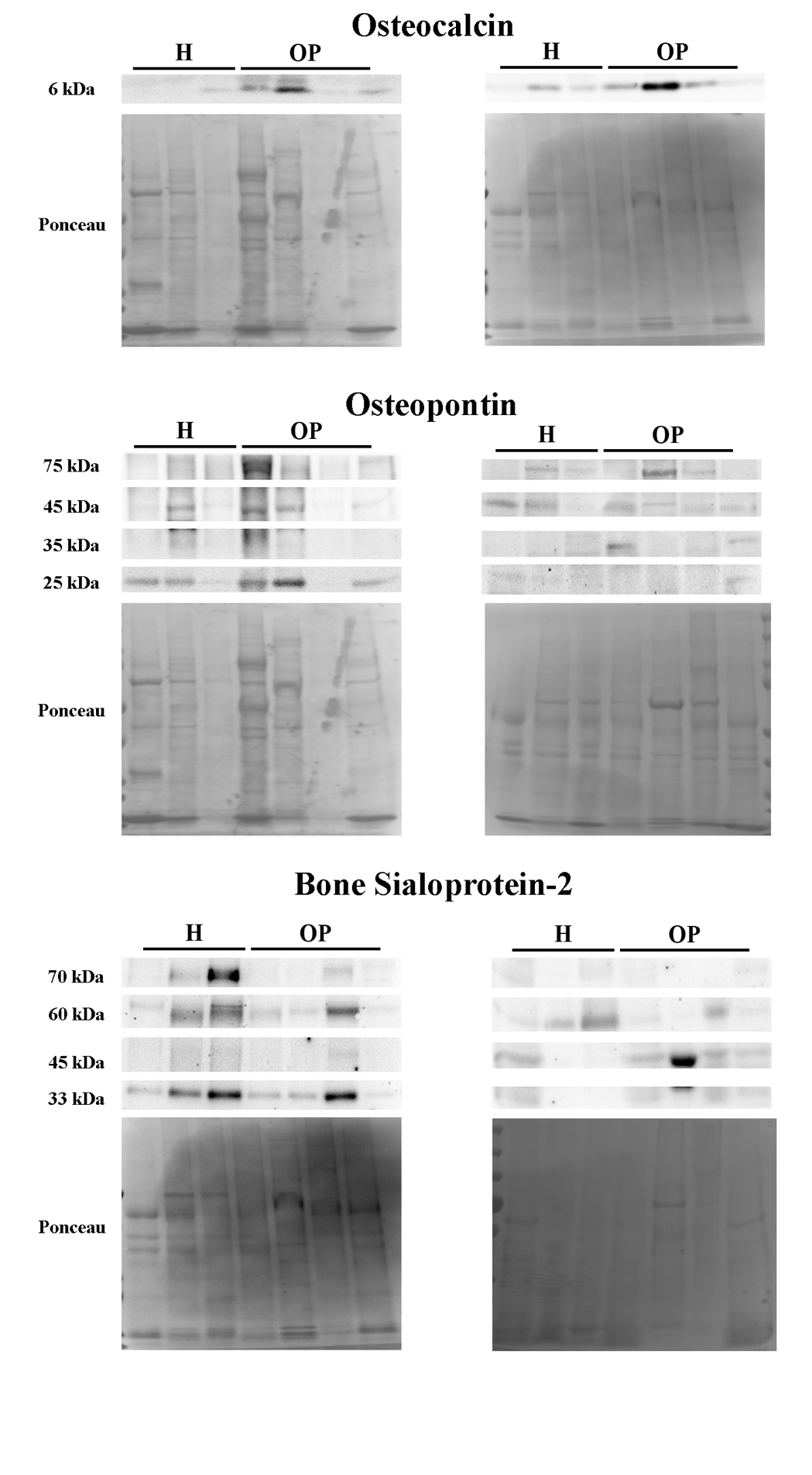


Figure 3S


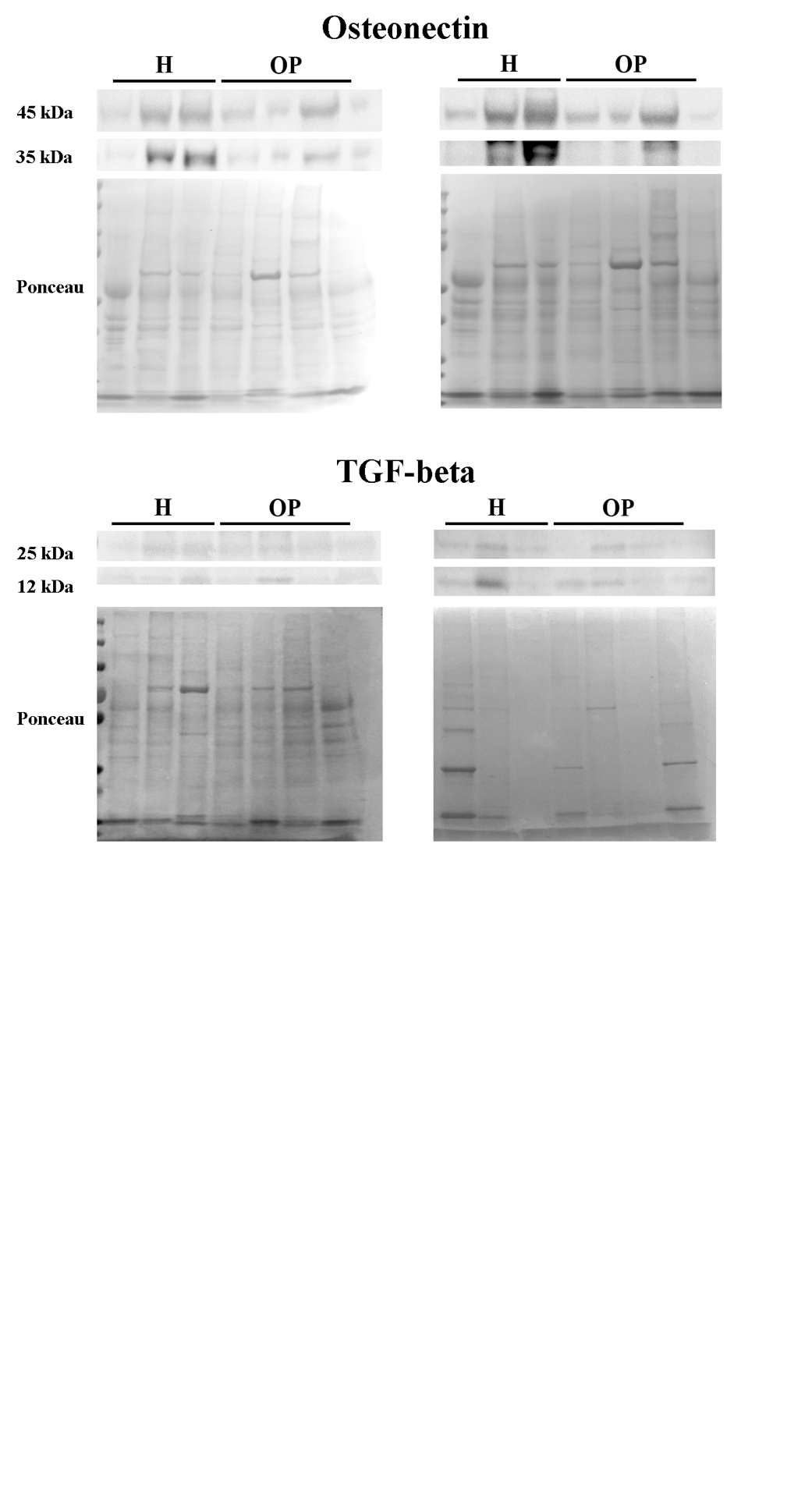

Supplement: Supplementary file 1 — Appendix S1 Supporting Information [file BIOF-48-1089-s001.docx]
